# Cellulosic Polymers for Enhancing Drug Bioavailability in Ocular Drug Delivery Systems

**DOI:** 10.3390/ph14111201

**Published:** 2021-11-22

**Authors:** Bharti Gupta, Varsha Mishra, Sankalp Gharat, Munira Momin, Abdelwahab Omri

**Affiliations:** 1 Department of Pharmaceutics, SVKM’s Dr. Bhanuben Nanavati College of Pharmacy, Vile Parle, Mumbai 400056, India; guptabharti643@gmail.com (B.G.); varshamishra369@gmail.com (V.M.); Sankalp.gharat@bncp.ac.in (S.G.); munira.momin@bncp.ac.in (M.M.); 2 Director(I/C), SVKM’s Shri C. B. Patel Research Centre for Chemistry and Biological Science, Vile Parle (West), Mumbai 400056, India; 3 The Novel Drug and Vaccine Delivery System Facility, Department of Chemistry and Biochemistry, Laurentian University, Sandbury, ON P3E 2C6, Canada

**Keywords:** ocular drug delivery system, hydroxypropyl methylcellulose, carboxymethyl cellulose, ethylcellulose, methylcellulose, hydroxyethyl cellulose, solubility, sustained release, bioavailability

## Abstract

One of the major impediments to drug development is low aqueous solubility and thus poor bioavailability, which leads to insufficient clinical utility. Around 70–80% of drugs in the discovery pipeline are suffering from poor aqueous solubility and poor bioavailability, which is a major challenge when one has to develop an ocular drug delivery system. The outer lipid layer, pre-corneal, dynamic, and static ocular barriers limit drug availability to the targeted ocular tissues. Biopharmaceutical Classification System (BCS) class II drugs with adequate permeability and limited or no aqueous solubility have been extensively studied for various polymer-based solubility enhancement approaches. The hydrophilic nature of cellulosic polymers and their tunable properties make them the polymers of choice in various solubility-enhancement techniques. This review focuses on various cellulose derivatives, specifically, their role, current status and novel modified cellulosic polymers for enhancing the bioavailability of BCS class II drugs in ocular drug delivery systems.

## 1. Introduction

The major challenges to developing ocular drug delivery systems are the presence of protective barriers, the complex anatomy, and pathophysiological functions of eye [1]. The lower absorption of drugs from conventional dosage forms such as eye drops is mainly due to rapid clearance of the drug with excessive lacrimal drainage and various ocular barriers [1]. A topically administered drug has less than 5% bioavailability in the anterior region and less than 1% bioavailability in the posterior region. Hence, a high dose of the drug is required to show the therapeutic effect, which in turn may cause toxicity. Frequent dosing also leads to low patient compliance [2,3]. The ocular system is a complex sensory organ with a size of 24 mm and a weight of 7.5 g, which is considered 0.05% of the human body [4,5]. The ocular system is classified into two regions: the anterior region, which includes the cornea, aqueous humor, conjunctiva, iris, ciliary body, and lens; and the posterior region, which includes the vitreous humor, retina, choroid, and the optic nerve [5].

**Figure 1:**
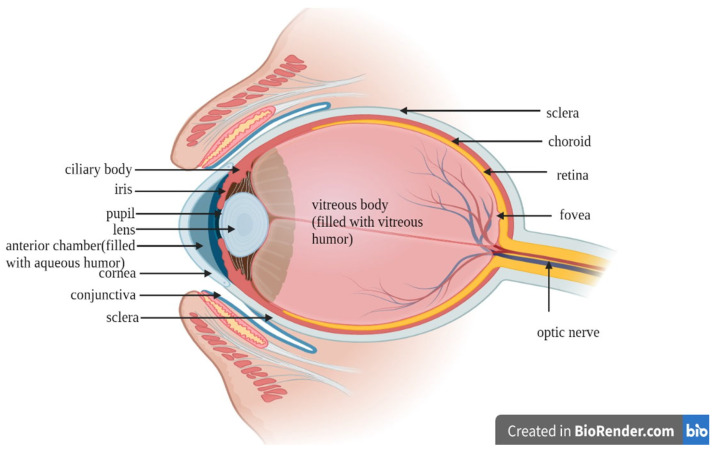
Anatomy of the eye.

To understand the fate of a drug in the eye it is important to understand the anatomy of the eye. The ocular system is made up of three layers: outer, middle, and inner. The outer region includes the cornea and sclera. The cornea is a physical barrier whereas the sclera holds the shape of the eye (Figure 1). The cornea has a vascular structure and contains sensory nerves that give the cornea transparency [6]. The negative charge on a cornea favors permeation of hydrophilic cationic drugs as compared to anionic drugs. The permeability challenges from the cornea is also due to the five layers that are present in the cornea, i.e., epithelial layer, bowman’s membrane, stroma, descemet’s membrane, and endothelium. The epithelial layer is a multilayer of stratified squamous epithelial cells that are connected by a tight junction and limit the hydrophilic drug penetration [4]. The stroma is charged and highly organized hydrophilic collagen, which further limits hydrophobic drug penetration [4]. The middle layer of the ocular system includes the iris, ciliary body, and choroid. The iris maintains pupil size and controls the quantity of light that enters the pupil. The ciliary body produces aqueous humor—a clear, slightly alkaline ocular fluid that supplies nutrients to the retina [6]. Three mechanisms are involved in the formation of aqueous humor: diffusion, ultrafiltration, and active secretion. Active secretion is the major contributor to aqueous humor formation. Around 70–90% of the aqueous humor leaves through the conventional path (aqueous humor passes through the trabecular meshwork, across the inner wall of Schlemm’s canal, into its lumen, and into draining collector channels, aqueous veins, and episcleral veins), whereas 10–30% leaves through the non-conventional pathway, which includes the ciliary muscle and supraciliary and suprachoroidal spaces [7]. Another important part of the eye is the conjunctiva, which is a thin, highly vascularized and semi-transparent connective tissue that covers the surface of the eyeball. It secretes mucous under the eyelids and extends to the corneal limbus. The conjunctiva plays an important role in maintaining the motion of the eyeball and eyelid due to its elastic nature [8]. The conjunctiva and sclera facilitate the absorption of large and hydrophilic drug molecules, whereas the nasolacrimal drainage absorption is facilitated by the pharynx, nasal mucosa, and GI tract [9]. The inner chamber of the eye is a vitreous chamber that is present behind the lens and filled by a gel-like material known as vitreous humor [3]. The lacrimal system contains the tear production system and the drainage system, which is an uninterrupted system that originates from the lacrimal puncta and proceeds from the lacrimal canaliculi to the lacrimal sac [10]. Another important anatomy includes the intraocular pressure of the eye, which is due to the presence of barriers such as the blood–aqueous and blood–retinal barriers, which may restrict the entry of the drugs into the intraocular chamber, resulting in loss of therapeutic efficacy of the drug [11]. To overcome these anatomical barriers, cellulosic derivatives offer their potential applications due to their properties such as sustained release, improve residence time, and controlled tunable drug release. Apart from these properties, cellulosic polymers are also used as tear substitutes due to their hydrophilic nature and water-retention capability.

This review focuses on challenges in the development of various ocular drug delivery systems, and the role of cellulose and their derivatives in addressing the solubility and bioavailability issue of BCS class II drugs.

## 2. Challenges in Ocular Drug Delivery System

**Figure 2:**
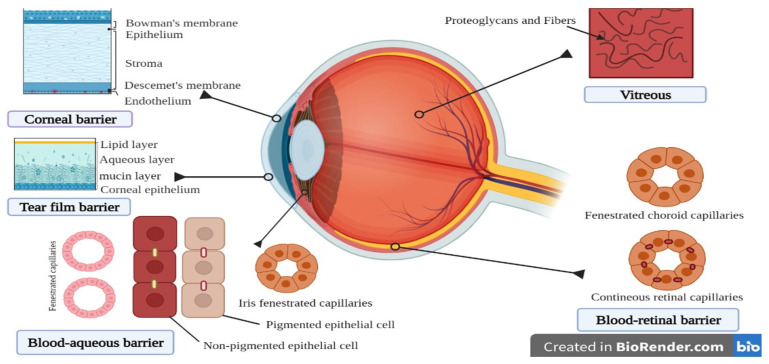
Anatomy of the ocular barriers.

The presence of protective barriers in the ocular system restricts the entry of drug molecules into it. The cornea (epithelial part) and conjunctiva (bulbar part) are major barriers on the outer surface of the ocular system (Figure 2) [2]. The nonionizable lipophilic drugs are distributed more in the corneal region, whereas ionizable lipophilic drugs get distributed in the aqueous humor [5]. The barriers found in the anterior and posterior regions of the body limit absorption, which in turn affects the bioavailability of many drugs [12].

The corneal and noncorneal routes are the two main pathways for intraocular absorption. The corneal route absorbs small and lipophilic molecules via the transcellular route and prevents the entry of hydrophilic drugs through the paracellular route. The noncorneal route includes the conjunctiva–sclera. The corneal route facilitates the absorption of hydrophilic drugs with higher molecular weights of 5000–10,000 Da, whereas the cornea and sclera allow passage of drug molecules with molecular weight less than 500 Da [5,9,13]. The influx and efflux transporters present in the cornea, conjunctiva, retina, and blood–ocular barrier region affect the transport of drug molecules. Modification of these transporters is one method for increasing bioavailability. Efflux transporters such as P-gp and multidrug-resistant protein (MRP) transport the drug out of the cell and decrease their bioavailability. The MRP efflux transporters transport the organic anions and their conjugates out of the cell [14]. Influx transporters transports drugs and the nutrients across the biological membrane. LAT1, LAT2, ASCT1, ASCT2, and B (0,+) are the amino acid-based influx transporters, whereas PEPT1 and PEPT2 are peptide-based influx transporters that play a major role in the influx of drugs in the corneal region [14].

Drug diffusion by the corneal routes have various ocular barriers, such as tear turnover, nasolacrimal drainage, corneal epithelium, iridial blood flow, trabecular, and uveo-scleral outflow. Whereas, the barriers for the diffusion through non-corneal routes are conjunctiva, sclera, mucus turnover, retinal pigment epithelium (RPE), and choriocapillaris [9]. Metabolites observed in the eye are because of the ocular metabolism or the hepatic or extrahepatic metabolism [15]. Ocular non-P450 oxidative and reductive enzymes are aldehyde oxidases, which are highly present in the ciliary body followed by the RPE, choroid, and iris. Xanthine oxidoreductase and xanthine oxidase are involved in the anterior regions’ microbial protection, whereas keto-reductase is found higher in the corneal epithelium. Ocular hydrolytic enzymes are esterases, which are located in the epithelium and stromal-endothelium of the cornea. Carbonic anhydrase is found in the iris-ciliary body of pigmented rabbits, and aminopeptidases M and A and dipeptidyl peptidase IV are found in the cornea of pig, rat, and humans. Cholinesterases are found in the retina and retinal pigmented epithelium of rats. The mono-acyl glycerol lipase enzyme found in the non-pigmented ciliary epithelium of mice plays a major role in increasing the intra-ocular pressure by the metabolism of endogenous 2-arachidonyl glycerol [16]. Apart from this, ocular barriers are generally classified as static and dynamic. The static barrier is also known as an anatomical barrier, which includes the cornea, sclera, conjunctiva, and retina. The dynamic or physiological barriers includes conjunctival blood flow, choroidal blood flow, lymphatic clearance, efflux transporters, nasolacrimal drainage, and tear turnover [2]. The corneal epithelial barrier allows the passage of lipophilic molecules but hinders the passage of hydrophilic drugs and those with molecular size larger than 10 A°. Another barrier for ocular drug delivery is the aqueous humor, which reduces the trans corneal diffusion [12].

Posterior barriers such as the sclera provide higher permeability for hydrophilic drugs than the cornea but restricts the entry of macromolecules. The retina limits the entry of larger drugs (>75 kDa). With aging, the Bruch’s membrane becomes thick, which decreases the transport of drug across the membrane and drains the lipophilic drugs into the blood circulation. The epithelial layer is also a major barrier that is connected by tight junctions, which limits the penetration of hydrophilic drugs. Stroma, which is composed of highly organized hydrophilic collagen, limits the entry of hydrophobic drugs [4]. The efflux transporters present on the blood–retinal barriers decrease the bioavailability of the administered drugs. The permeation of drug through the blood–aqueous barrier depends on the osmotic pressure and nature of the drug molecule [12].

## 3. Routes of Ocular Drug Delivery System

There are three routes of ocular drug delivery: topical, local, and systemic [17]. The ideal route of administration of drugs is determined by the region of the eye to be treated. Topical treatment usually works effectively on the conjunctiva, cornea, anterior chamber, and iris [18]. As most topical formulations do not enter the posterior region, local injection or systemic treatment is often required.

### 3.1. Topical Route

The topical route is preferred for management of anterior segment diseases, due to their low cost, ease of administration, and patient compliance [19,20]. However, this route is not able to deliver the drug to the posterior segment due to the anatomical and physiological barriers of the eye. This route is applied for the administration of eye drops, ointments, and gels that are used for anterior segment diseases. However, the topical route has several disadvantages, such as less contact time, low permeability, and faster elimination of the drug [21]. The high tear turnover, nasolacrimal drainage, and tear dilution results in loss of 90% of the topically administered drug [22].

### 3.2. Local Route

Periocular routes include delivery of the drugs through the sub-conjunctival and retro-bulbar regions. Through this route, the drug is delivered to the external surface of the sclera, thereby decreasing the risk of endophthalmitis and retinal damage, which is observed in the intravitreal route [23]. In the subconjunctival route, the formulation is injected in the area below the conjunctival membrane, the drug bypasses the conjunctival-cornea barrier and directly enters the sclera. The sclera has low resistance to penetration of drugs and has less or negligible protease activity as compared to the cornea. In the retro bulbar route the formulation is injected into the eyelid and orbital fascia for deposition of the drug behind the globe in the retrobulbar space [17]. However, this route is not much preferred as it may damage the orbital structure of the optic nerve [24,25]. The sub-retinal route is used to deliver the drug directly to the outer retina for management of the retinal degenerations, which originate in the photoreceptors and RPE [26,27]. It is a highly invasive method and causes ocular damage such as lesions in RPE, sub or pre-retinal fibrosis, hemorrhage, and retinal detachment [25]. Intravitreal route is the main route that delivers large molecules to the posterior region of the eye [28]. It can deliver formulations of up to 20 to 100 µL; however, reapplication of a local anesthetic is required. Intravitreal injection creates complications, such as retinal detachment, endophthalmitis, intraocular hemorrhage, and uveitis cataract [29]. Furthermore, the intra-cameral route is used for direct delivery of the drug to the anterior chamber; however, general anesthesia is required before injection. This route may cause damage to intraocular structures such as the iris, lens, and corneal endothelium [25,30].

### 3.3. Systemic Route

The systemic route is used to treat the diseases of the posterior segment, which are difficult to treat by the topical route [31]. However, the systemic route has some drawbacks, such as the need for a high dose and frequent dosing due to drug dilution in the blood, blood ocular barriers, and low cardiac output to the eye. In the systemic route, the drug undergoes metabolism by the liver and clearance by the kidney, which causes less of the drug to reach the vitreous humor. [14].

## 4. Conventional Ocular Drug Delivery System

There are various conventional ocular drug delivery systems on the market, such as eye drops, ointments, emulsion, suspension, and polymeric gels [32,33]. The eye drops makes almost 70% of the prescribed dosage form for eye treatment due to its advantages such as patient compliance, drug efficacy, cost-efficacy, non-invasive, safe, and ease of bulk manufacturing of the formulation [34,35] Only 20% of the total inserted eye drop dose is retained in the precorneal region because of the blinking effect loss and excessive lachrymal fluid secretion. [35]. The bioavailability of the drug from eye drops is limited due to dilution and loss of the drug due to tear drainage and the eye pocket size, which has a low liquid holding capability [36]. The loss of the drug due to the abovementioned reasons leads to multiple administration, leading to patient non-compliance [37,38]. One of the strategies to improve the residence time of the solution is to increase the viscosity of eye drops, thereby improving the bioavailability of the drug. Polymers such as hydroxypropyl methylcellulose (HPMC), carboxymethyl cellulose (CMC), hydroxypropyl cellulose (HPC), hydroxyethyl cellulose (HEC), and poly alcohols have been extensively explored for improved residence time and bioavailability of the drug given in the form of eye drops. Further, the permeation enhancers such as cyclodextrins improve the uptake of the drug and the solubility of the hydrophobic drug. However, it is important that the selection of additives for modification in the drug delivery system is done very carefully as the eye is a highly sensitivity organ [32,35].

Other conventional formulations used for ocular delivery are emulsions and suspensions [39]. Patel et al. in their study showed that emulsions can increase the solubility and bioavailability of ocular drugs [35]. The oil in water (*o*/*w*) type of emulsion is generally preferred because of its better tolerance and low ocular irritation [40]. Liang et al. proved that an emulsion enhanced the corneal permeation, precorneal residence time, sustained drug release, and bioavailability of the drug in male albino rabbits [41]. However, the conventional emulsions have several disadvantages, such as being less stable and prone to instabilities such as coalescence, flocculation, and creaming, and also destabilization by the tear fluid [32]. Suspensions are advantageous over eye drops (solutions) as they increase the contact time of the drug and duration of action because of the dispersed insoluble drug that cannot be washed away by the dilution of the tear [42]. Conventional suspensions do not have a uniform size distribution, which increases the duration of action. Smaller drug particles in the suspension absorb quickly while the larger particles retain and dissolve slowly in the precorneal section [42]. Despite these advantages, suspensions have several disadvantages, such as the need to shake before use and variation in the dose, which may reduce the drug accuracy [32]. To overcome these problems, ointments are used. Because of their viscous nature, they do not get washed away by the tear fluids, unlike the liquid preparations [43]. As the viscosity of the ointments is high, it causes blurring of vision [37,44]. Polymeric gels are made up of mucoadhesive polymers to increase the contact time of the formulation. Mucoadhesive polymers are generally used to enhance the efficacy of the formulation as these polymers adhere to the biological tissues and increase the contact time and bioavailability [45,46]. Polymeric gels are of two types: in situ gelling systems and preformed gels [46]. In situ gels are preferred over preformed gels as these formulations act smartly by changing the viscosity in the site of application [47].

Presence of barriers and defense mechanisms, such as a high tear turnover rate, dilutes the efficacy of the conventional systems. Therefore, conventional formulations requires a high concentration of drugs, which may cause local cellular damage and other systemic adverse effects that decrease the efficacy of the treatment [9].

## 5. Novel Ocular Drug Delivery

Apart from advantages, conventional formulations suffer from some disadvantages such as a short retention time, leading to rapid tear clearance and nasolacrimal drainage, resulting in low ocular solubility and bioavailability (that is, ˂5%) [48,49]. This disadvantage leads to the development of novel ocular drug delivery systems such as nanoparticles, nano-micelles, liposomes, contact lenses, inserts, implants, and microneedles [48].

### 5.1. Nanoparticles

A nanoparticle is defined as any particle that has a diameter range of 1 to 100 nm [50] and is made of natural and synthetic lipids, polymers, phospholipids, or metals. Nanoparticles are of two types, such as a nano-capsule and nanosphere [51]. The drug is encapsulated into a polymeric capsule in the nano-capsule whereas the drug is uniformly dispersed throughout the polymeric matrix in the nanosphere. One of the approaches of the nanoparticle is solid lipid nanoparticles [52] as it has certain advantages, such as enhancing corneal absorption and corneal bioavailability for both types of drugs—hydrophilic and hydrophobic; providing autoclave sterilization of the formulation; and does not have toxicity, as the lipids used are physiological at the time of preparation. SLN also has a sustained-release activity; for example, tobramycin SLN shows a six-hour sustained release compared to tobramycin eye drops of the same dose [32]. Yan et al. prepared a nanoparticle for ocular delivery for the treatment of ocular wound healing by the use of cellulose nanofibrils and poly lactic acid [53].

### 5.2. Nanomicelles

Nanomicelles are the commonly used carrier to deliver the drug to the clear aqueous solution. It is made of surfactants or polymers that are amphiphilic in nature, and have the property to self-assemble themselves in the micelle form [54]. Micelles are of three types: regular, reverse, and unimolecular. Regular micelles self-assemble in an aqueous medium whereas reverse micelles self-assemble in a non-aqueous medium, and both are copolymers [55]. Unimolecular micelles are block copolymers that have a hydrophilic and hydrophobic site in them. The advantage of nanomicelles are their easy preparation method, improve drug solubility, increase penetration into tissue, low toxicity, and targeted delivery [32]. Mehra et al. developed copolymer-based nanomicelles for delivery of Everolimus for the treatment of uveitis by using a grafted polymer of polyvinyl caprolactam–polyvinyl alcohol–polyethylene glycol (PVCL-PVA-PEG) [56].

### 5.3. Liposomes

The drug is encapsulated in the liposome and is delivered as an eye drop [57]. Natarajan et al. prepared a latanoprost-loaded egg phosphatidylcholine liposome. A liposome is stable for 6 months when stored at 4 °C and it is stable for 1 month when stored at 25 °C. The 60% latanoprost release was slow and sustained for two weeks in vitro; a more sustained IOP-lowering property is seen in liposome formulations compared to topical latanoprost [58,59]. Blazaki et al. developed a novel liposome aggregate platform system for calcein, FITC-dextran-4000, and flurbiprofen, which is encapsulated in a negatively charged liposome; this study showed that liposome is one of the most promising and safe approaches for ocular delivery [60].

## 6. Various Approaches for Drug Delivery

Novel formulations are incorporated into these various approaches, such as contact lenses, implants, microneedle, etc., for sustaining the drug release [35].

### 6.1. Contact Lenses

The first contact lenses were developed for glaucoma in 1974 by soaking vinyl pyrrolidone or acrylic co-polymer contact lenses for three days in 1% pilocarpine eye drops. Some of the studies show that soft contact lenses give a sustainable drug release and they are transparent, and therefore do not impair vision [61]. Soft contact lenses were made of the cross-linking of a hydrogel with a water-soluble polymer [62]. Contact lenses can be loaded with vitamin E to improve the drug release. Five marketed silicone contact lenses are ACUVUE ADVANCE and ACUVUA OASYS by Johnson and Johnson vision care; O2OPTIX by Alcon, Fort Worth; NIGHT and DAY by Alcon, Fort Worth; and pure vision by Bausch and Lomb, Bridgewater; these were used to increase the release of dexamethasone [62]. Kang et al. developed contact lenses of oxidized hydroxyethyl cellulose and an allyl co-polymer-based hydrogel [63].

### 6.2. Ocular Inserts

The first ocular insert reported is a small portion of filter paper impregnated with drug solutions such as pilocarpine HCL and atropine sulfate [64]. The ocular insert was of three types: soluble, insoluble, and bio-erodible. The soluble and bio-erodible differ in their underlying chemical processes [65]. The polymers used for the formulation of inserts are methyl cellulose (MC), hydroxypropyl methylcellulose (HPMC), ethyl cellulose (EC), polyvinyl pyrrolidone (PVP), polyvinyl alcohol (PVA), chitosan (CS), and gelatin [62]. The shape of the insert is the major challenge for the preparation of inserts, as the shape of the insert affects the capacity of drug loading, comfort, and retention time. The human volunteers show that rod-shaped inserts are well tolerable [66]. Marketed ocular inserts are ozurdex, surodex, iluvien, mydriasert, retisert, and lacrisert, and others are in clinical trials [67]. Franca et al. developed chitosan/hydroxyethyl cellulose inserts for delivery of dorzolamide for the treatment of glaucoma [68].

### 6.3. Intraocular Implants

Intraocular implants are inserted into the eye by a surgical process, and drug release is extended for a long period. The implants are not biodegradable so there is a need to remove the implant by surgery, which has many risks with this type of delivery system. However, in biodegradable polymeric implants, there is no need to remove the implants by surgery [51]. Implants can also be made as stimuli-responsive delivery systems, but the currently marketed implants extended the release but do not change the rate of the drug release. There are many ongoing studies related to stimuli-responsive polymeric implants [37,69,70,71]. Felipe et al. prepared an implant for the delivery of bimatoprost for treatment of glaucoma and ocular hypertension; in a 12-week study, they showed that a bimatoprost implant was not inferior to timolol and it has potential for improved adherence and decrease the treatment of glaucoma [72].

### 6.4. Microneedles

Microneedles are one of the delivery systems; they are made of metals or polymers and have a length of 15–1500 µm, thickness of 1 to 25 µm, and width of 50 to 250 µm [73]. It is less invasive than the injection due to the micron dimension of the device and also provides a targeted release [74]. Jiang et al. used a microneedle of 500 to 750 µm to deliver pilocarpine by intrascleral route in the anterior region. They found an increase in absorption of 45 fold compared to eye drops [59]. Roy et al. developed a microneedle patch containing liposomal or free amphotericin-B as a treatment for fungal keratitis because it is a less invasive delivery system compared to ocular injections [75].

## 7. Strategies for Enhancing Ocular Drug Delivery System

To overcome the disadvantage of topical ocular delivery, researchers have given two strategies, such as enhancing the corneal residence time by the use of a viscosity enhancer, in situ gel, and a mucoadhesive agent [76]. Some delivery systems give a prolonged retention time with a decreased frequency of drug dosing. A low viscous preparation has more patient compliance; however, enhancing the viscosity of the formulation increases the retention time and improves the bioavailability of the drug [76]. Natural and synthetic polymers and biopolymer are used due to their viscosity-enhancing activity [44]. These polymers cause a slower elimination of the drug; examples are cellulose derivatives such as protein (collagen, silk, gelatin), polysaccharide (chitosan, starch, alginate), polyesters (polycaprolactone, polylactide, polylactide/polyglycolic copolymer) [35], and cellulose derivatives such as HPMC [77], hydroxyethyl cellulose [78], and methylcellulose. In situ gels are delivered as a solution or suspension and rapidly undergoes sol-gel transition [79] upon external stimuli such pH, temperature, and ionic strength [80]. This has merits such as reproducible and accurate dosing. Low vision impairment, being easy to administer, and a prolonged residence time decreases the frequency of administration and has low nasolacrimal drainage. Depending on the physiological mechanism, the three categories of polymers are (1) pH-triggered in situ gels: this polymer has weakly basic and acidic groups which accept and release protons in response to a pH change. (2) Temperature-triggered in situ gel, the low critical system temperature (LCST) is the phase-transition temperature; below this point the hydrogen bonds between the polymer and water molecules increase the dissolution of the polymer, but above this point, when the temperature increases, the hydrogen bonds breaks and a hydrophobic interaction appears that is a sol-gel transition. Finally, (3) ion-triggered in situ gelling polymers, because of the mono and divalent cation in tears, cross-linking of the sensitive polymer occurs [81]. The mucoadhesive agent adheres to the mucous membrane; this adhesion enhances the retention time of the drug and controls the drug release along with enhancing the bioavailability and more patient compliance [82]. The mechanism of muco-adhesion is the contact phase and the consolidation phase. The former involves the contact between the agent and mucus that causes the spreading and swelling of the preparation. In the latter phase, the mucoadhesive polymer is activated in the presence of moisture, which causes molecules to break freely and be joined by the force of van der Waals and hydrogen bonding [83].

Secondly, by increasing the corneal permeability, through the use of prodrugs, penetration enhancers, and a colloidal system, such as nanoparticles and liposomes [84]. Prodrugs are inactive substances; they need to be transformed chemically or enzymatically [85]. The active compound has carboxyl or hydroxyl groups that are esterified to give a lipophilic substance. The corneal epithelium, esterase, is 2.5-fold more as compared to the endothelium and stroma [86]. An example is the nepafenac prodrug, used for inflammation and the pain associated with cataract surgery [87]. This shows that lipid vesicles are compatible with the corneal epithelium and leads to enhanced solubility and ease of transportation, across the cornea. Permeation enhancers can improve drug permeability in the corneal epithelium, reducing the corneal barrier resistance. Permeation enhancers decrease the dose size and enhance the bioavailability [88]. For example, Benzalkonium chloride alters the ocular barrier, but it has a toxic effect when used for several days by accumulating in the cornea [89] and EDTA acts by altering the tight junction of superficial cells and cause paracellular transport of the drug. Cyclodextrin, by complexing with the drug, solubilizes the lipophilic molecules [90] and results in increased permeation [91]. The colloidal system provides controlled drug release and prolonged pharmacological effects. On localized retention in a cul-de-sac, the drug can be delivered under external stimuli such as light or by diffusion. NP can overcome the ocular barriers, maintaining an optimal concentration and drug permeability [92]. Liposomes are large in size as they contain electrostatic attraction between the positive and negative charges of polymers and phospholipids [93].

Topical formulations are the most widely used treatment approach for ocular drug delivery. The vehicles or bases of topical formulations, such as a solution, suspension, or ointment, are very critical in determining the drug delivery to the eye. Vehicles should not irritate the eye and should be compatible with the rest of the ingredients as well as the packaging material [94]. Ophthalmic solutions and suspensions have purified water or most preferably sterile water for injection as their vehicle. In the case of topical ointment, a mixture of white petrolatum and liquid paraffin is the most widely used vehicle. However, because of the advantages of water-soluble bases over petrolatum bases, such as better spreadability, pH, lubricity, stability, and low irritability, the use of water-soluble bases, such as gels containing PEG 200, PEG 400, carboxymethylcellulose, and Carbopol, has increased. It is necessary to increase the viscosity of the formulation to get a prolonged residence time and improved bioavailability [95,96]. Vehicles containing viscosity enhancers, such as CMC, MC, HPMC, and HMC, improve the retention time of topical ophthalmic formulations. Vehicles containing mucoadhesive polymers interact with the mucin layer and increase the residence of the formulation while also successfully increasing the bioavailability [97]. In all ophthalmic formulations, sterility is an absolute requirement. Various sterilization methods, such as an autoclave, dry heat, membrane filtration, ethylene oxide, gas plasma, and irradiation, can be employed, depending on the feasibility and thermal stability of the formulation. Most of the formulations are terminally sterilized by either dry heat method, autoclave, or irradiation. In the case of a liquid vehicle, the formulation can also be sterilized by filtering it through a 0.22micron membrane filter in a sterile container [98]. Aseptic preparation involves pre-sterilization of the vehicles or ointment bases and all the ingredients involved in the formulation. The production takes place in a clean room. It is convenient to perform terminal sterilization rather than an aseptic production process [98,99].

The antimicrobial preservative is a very crucial component of the ocular drug delivery system. Preservatives should be chosen based on properties such as efficacy against a wide range of organisms at extremely low concentrations, compatibility with packing components, long shelf-life, stability, and solubility [100]. The quaternary ammonium compounds, such as benzalkonium chloride, 2-poly(ethyl alcohol), chloro-butanol, and parabens, are commonly used preservatives that meet most of the criteria discussed above [101]. The presence of preservatives in ocular formulations is considered to be the cause of epithelium damage but they are necessary, especially in a multi-dose container. Preservative such as benzalkonium chloride is well known for causing ocular cytotoxicity, therefore some newer preservatives such as Polyquaternium-1, sodium perborate, and stabilized oxy chloro-complex are being explored. In some instances, a preservative-free single-dose container was used, usually in the case of patients with serious allergies or surgical conditions. Preservative-free containers need to be very carefully sterilized and stored to avoid bacterial growth [102].

## 8. Cellulose and Its Derivatives in Ocular Drug Delivery System

Cellulose is one of the most widely used polymers in ophthalmic formulations. In the 1940s, methylcellulose (MC) was first used in ocular formulations as a viscosity enhancer. Since then, cellulosic polymers have been widely studied in animals, as well as in humans, for ocular administration [103]. As pure cellulose is insoluble in water, various cellulosic derivatives are employed extensively in ocular formulations. The cellulosic derivatives that are most commonly used in ocular formulations are methylcellulose (MC), hydroxyethyl cellulose (HEC), hydroxypropyl cellulose (HPC), hydroxypropyl methylcellulose (HPMC), and carboxymethylcellulose (CMC) [104]. Cellulosic derivatives have valuable viscosity-increasing properties, which are very useful in polymer-based ophthalmic formulations for improved bioavailability. These macromolecules also have evident potential as a carrier in ocular drug delivery. Additionally, the swelling properties, chemical properties, and structural morphology of these derivatives influence the release mechanism of the drugs loaded in these systems to a great extent [105,106,107]. It can be obtained from various natural sources, such as vegetables, cotton fiber, woods, and even from marine animals such as tunicates, as well as found in bacteria such as algae, fungi, and invertebrates, or may even be synthesized in labs [108,109]. The extensive production of cellulose, which is 7.5 × 10^10^ t annually, shows that there is an abundant reservoir of this polysaccharide, which helps in reducing the overall cost of the formulation [108,110]. Cellulose, as a raw material, is suitable for the large-scale manufacturing of various products. Cellulose can be altered easily utilizing chemical reactions; therefore, many derivatives of cellulose are produced for application in various ophthalmic preparations. It can be further modified to meet specific requirements [110]. Easy accessibility and the valuable properties of cellulosic polymers have made them a very attractive choice of polymer for ophthalmic formulation.

Cellulose is a sustainable natural polymer in the world and it is a primary component of plants [108,110]. It has very good mechanical properties that give strength to plants [108]. In the past few decades, the development and innovation of various delivery systems in formulations, science, medicine, and technology brought forward the application of this natural molecule globally [111]. Production of cellulose is 7.5 × 10^10^ t annually, showing that there is an abundant reservoir of this polysaccharide [108,110]. Cellulose is suitable for the large-scale manufacturing of various chemicals and products as a raw material. Cellulose can alter, easily utilizing chemical reactions, and therefore many derivatives of cellulose are produced for application in various preparation. It even can be modified to meet the various properties [110]. It can be obtained from various natural sources such as vegetables, cotton fiber, woods, and even from marine animals such as tunicates, and also found in bacteria such as algae, fungi, invertebrates, or may be synthesized in labs [108,109].

Cellulose was discovered by the French chemist Anselme Payene in 1838 [109]. Cellulose is a high-molecular-weight homopolysaccharide and composed of β-1,4-anhydro-D-glucopyranose units, which is linked to an acetal molecule by covalent bonds between the C4 of the hydroxyl group and C1 of carbon. Anhydro glucose molecules contain one primary and two secondary hydroxyl groups [95,112]. This hydroxyl group forms the hydrogen bonds that are inter and intramolecular bonds; because of the very strong bond, cellulose is not soluble in aqueous and organic solvents [108,113]. Two glucose moieties are linked through the β-1-4 bond and forms cellobiose units [109], with a high molecular weight (162.14 g mol^−1^), and the degree of crystallinity makes cellulose aqueous insoluble. The hydroxyl group of D glucose is the favorable site for modification and to form different derivatives [114].

## 9. Properties of Cellulosic Polymers

Cellulose can be modified by esterification or etherification methods; this modification can lead to water solubility, viscosity enhancer, water binding ability, adhesiveness, film former, thickening agent, swelling, and emulsifying properties. These properties of modified or derivatives of cellulose lead to its wide applications [115,116]. Cellulose derivatives are preferred over cellulose because of their aqueous and organic solvents solubility [109]. Cellulose derivatives are green molecules, have a low cost, and have very good properties such as a high chemical stability and good solubility, as well as having a very good biological affinity, moldability, porosity, and are physiologically safe molecules [111,117]. These molecules also have good biodegradability and are biocompatible; for example, carboxymethyl cellulose degrade in a few days in the presence of enzymes such as cellulase [109,117]. Bacterial cellulose has different structural features and properties, such as a high crystallinity up to 70 to 80%, high purity, high degree of polymerization (8000), high water content (up to 99%), and a higher mechanical stability than natural cellulose [118]. Cellulose ethers can be produced in varying properties, such as water retention capacities, film formation ability, surface activity, and pseudo-plasticity; due to these properties cellulose ethers are used in various food products, cosmetics, pharmaceuticals products, 3D printing, and other products [117]. Cellulose molecules can be used as a control release system; the most commercially available ones are sodium carboxymethyl cellulose, methylcellulose, and hydroxypropyl methylcellulose, which has less crystallinity and a smaller crystal size, but have a large pore size and thermal stability [111,113]. It has 29 °C as the lower critical solution temperature (LCST), which shows that at a low temperature it acts as a Newtonian flow and increases in viscosity is seen above 29 °C [113]. Cellulose derivatives can be used for different purposes, such as in hemodialysis, biosensor [119], textile fibers [120], nanoparticles [121], and hydrogels [122]. Therefore, cellulose derivatives are widely used to enhance the bioavailability of various class II drugs, by increasing the aqueous solubility of the drugs in the ocular system.

## 10. Cellulosic Polymers in Ocular Drug Delivery

Cellulose derivatives are divided into two parts: cellulose ether is the water-soluble derivative, which include carboxymethyl cellulose (CMC), hydroxypropyl methylcellulose (HPMC), methylcellulose (MC), ethyl cellulose (EC), hydroxypropyl cellulose (HPC), and hydroxyethyl cellulose (HEC); and cellulose ester is the water-soluble or insoluble derivative, such as cellulose acetate (CA) and cellulose acetate phthalate (CAP) [117,123] (Table 1). These derivatives have more solubility, suitable hydrophilicity or hydrophobicity, viscoelasticity, and thermal stability [123].

### 10.1. Cellulose Ether Derivatives

#### 10.1.1. Methylcellulose

Methylcellulose is a water-soluble, non-toxic, tasteless, and odorless cellulose derivative (Table 2). It exhibits sol-gel phase transition that is temperature-sensitive (LCST polymer) [131], and the mechanism of gelation is a hydrophobic interaction between the molecules that contain a methoxy substitution [103]. Various thermo-responsive ocular drug delivery can be made by use of phase transition, and the critical gelling concentration depends on polymer–polymer interactions, polymer–solvent interactions, hydrophilic–lipophilic characters, molecular weight, and the flexibility of a chain. Gelation can be affected by a high concentration of electrolytes, surfactants, sugar, and natural gums; this decreases the polymer hydration and gelation temperature [126]. As the concentration of the MC increases, the gelation temperature decrease linearly [132].

Yidan Wei et al. studied the in-situ gel of Betaxolol hydrochloride, which is used as a model drug, poloxamer 407, where methylcellulose was used as the carrier. In this study, a thermosensitive in situ gel was formulated and evaluated. In vitro study shows that incorporation of HPMC 606W (4%) into poloxamer 407 (15%) and PEG 4000 into MC (2%) gives a significant gelation temperature and a sustained release the same as the BH eye drop; the in vivo study also shows the same drug concentration in cornea, iris ciliary, and aqueous humor. The concentration of methylcellulose increases the gelation temperature around 45 °C, because as the concentration increases, the hydrophobic interaction of the MC increases, causing a decrease in the gelation temperature. It is not fit for ocular delivery. PEG is hydrophilic and leads to an increased hydrophobic interaction by acting as a proton acceptor; therefore, in this study, they kept the MC at 2% constant, and for a different proportion of PEG. PEG decreases the extension of MC in water. A large MW PEG (more than 1500) is long enough to decrease the extension of MC, and therefore PEG 1500/4000/6000 (5%) had a gelation temperature of around 34 °C, which is suitable for ocular delivery [132]. Another study related to MC was done by Xia et al., where needle clogging by microparticles is a challenge for injectable ocular delivery. In this study, hyaluronic acid (HA) and MC were used because they are applicable for ocular injection; this polymer was blended with Sunitinib malate-loaded PLGA microparticles (MPs); this polymer has shear thinning viscous properties, which eases the injection by a fine-gauged needle. HA and MC decreases the burst release and extend the release of the drug from the microparticles. The particles were entrapped in the HA and MC mesh-like network as this polymer has sufficient viscosity for retention of microparticles for a prolonged period. The physical adhesion or attachment to the conjunctiva may also extend the release of drugs [133]. A similar study was done by Nagai et al.; in this study, an in-situ gel was prepared by assimilating TL-NPs and the methylcellulose (0.5–3%) to get the prolonged residence time of the drug. The drug is more dispersed in the formulation with MC, and diffusion decreases as MC are added. An in vivo study shows the TL quantity increase in lacrimal fluid. An optimal amount of MC (0.5–1.5%) increases the TL in the cornea and conjunctiva, and the anti-inflammatory effect of the drug was seen. As the concentration of MC (3%) is in excess, the anti-inflammatory effect was reduced compared with the TL-NPs formulation of MC (0.5–1.5%). Therefore MC (0.5–1.5%) has a prolonged residence time in the pre-corneal and pre-conjunctival part of TL [134]. Wafa et al. also studied increasing the residence time of Pilocarpine nitrate drug, an in-situ gel/film-forming system. In this study, they used polyvinyl alcohol (PVA) as a film former, sodium alginate as bio-adhesive, and the effect of CMC, MC, Carbopol, and PVP was studied. All inserts had significant bio-adhesion in the in vitro study, and showed the matrix diffusion kinetics of the formulations [135].

#### 10.1.2. Hydroxypropyl Methylcellulose

HPMC is also called Hypromellose and it is a white or pale white cellulose used for a different purpose. It is a hydrophilic derivative used in oral and oro-mucosal drug delivery as a controlled release system; it can swell and form a gel, is stable in the pH range of 3–11, and cannot be cleavable by a gastric enzyme [124,136]. HPMC, which is produced by a hydroxyl group of cellulose, is partially etherified by a methyl group and a small quantity is substituted by hydroxypropyl groups. This is the most widely used polymer among the different derivatives of cellulose as it is a biodegradable [137] and biocompatible material [138,139], and has transparency and rheological properties [140] (Table 3). HPMC is a semi-synthetic dietary fiber of cellulose; it is a carbohydrate containing anhydrous glucose units. It is used as a prolonged release excipient [139] emulsifier, thickener, stabilizer, and forms a viscous solution [141] when it come into contact with water or GI fluid [142]. It is inert to many drugs and is used in capsule form [143] and has a good encapsulation efficacy and has the option of 3D printing [144]. It is used widely due to regulatory acceptance, ease of manufacture, and preparation of the control release formulations [145]. HPMC-based ongoing clinical trials are mentioned in Table 4 HPMC has negative thermo-sensitivity, it has a lower critical solution temperature (LCST) of 50 °C. Below this temperature, HPMC is soluble in water, and above it is not soluble when gelation takes place. However, on the eye surface, the temperature is less than LCST, and thus has less effect on viscosity [146,147]. HPMC K4M and HPMC E4M possess shear thinning properties [144]. HPMC is produced by various companies and is available in several trademarks such as METHOCEL (Dow Chemical Company, Rochester, NY, United State of America (USA)), BENECEL (Ashland, Rotterdam, The Netherlands), METOLOSE, PHARMACOAT (Shinetsu Chemical Company, Tokyo, Japan), and AFFINISOL (Dupont, Brooklyn, NY, USA) [124]. This polymer has a sol-gel phase transition that is a temperature-sensitive LCST of the polymer. The gelling concentration of various cellulose derivatives depends on the polymer–solvent interaction, polymer–polymer interaction, molecular weight, the flexibility of the chain, and other characteristics of polymers. Gelation is affected by the presence of a high concentration of electrolytes, sugar, natural gums, and surfactants; this decreases the hydration of the polymer and indirectly by the salting-out process due to the gelation temperature [126]. The transition temperature of HPMC is 75 to 90%. The gelation temperature can be lower up to 40% by the addition of sodium chloride by lowering the hydroxypropyl molar substitution [148]. Minitablets, which are used in the eyeball, are made of various polymers, e.g., cellulose derivatives such as HPMC, CMC, and HEC [94].

HPMC is used for enhancing the bioavailability by increasing the penetration of hydrophobic drug molecules [149]. Liu et al. examined the ocular delivery of Fk506 loaded nanomicelles in an amino-terminated polyethylene glycol block poly (D, L)-lactic acid and HPMC, which shows enhanced bioavailability and efficacy of FK506 (tacrolimus) for ocular disorder therapy in anti-allograft rejection; the nanomicelles were prepared by solvent evaporation-induced self-assembly in an aqueous medium and have a diameter of 101.4 ± 1.3 nm. These nanomicelles that solubilized the drug were evaluated for their stability, drug loading, encapsulating efficacy, surface tension, and in vitro release of the drug, and results show that the nanomicelles were more suitable for intraocular then the 0.05% suspension drops of the same. In vitro studies show a significantly high quantity of FK506 permeated and an in vivo study shows an increased concentration and prolonged retention of FK506 [150]. Elkasabgy et al. explored the potential of HPMC as a precipitation inhibitor. The solubility and bioavailability of econazole was increased by preparing an ocular supersaturated self-emulsifying drug delivery system (S-SNEDDS) using various oils, surfactants, and co-surfactants. Globule size, polydispersity index, and irritation potential was tested by using Hen’s Egg Test-Chorioallantoic membrane (HET-CAM). The precipitation inhibitor effects were studied in an in vitro precipitation test of S-SNEDDS, and permeation of econazole was studied in rabbits. The in vitro results show the use of HPMC sustains the S-SNEDDS release by inhibiting econazole precipitation; this shows high Cmax, Tmax, and AUC08, and successful formulation with improving bioavailability [151]. Nanda et al. also work on the permeation enhancement of mucoadhesive ocular film by the use of HPMC. He worked on the corneal permeation improvement of amlodipine anti-inflammatory drug and the effect of sulpho butyl-ether β cyclodextrin, and tested these on a carrageenan-induced rabbit model. Maximum swelling and higher erosion were seen in the film without cyclodextrin. A big improvement in the drug release and permeation was seen in sulfo butyl ether β-cyclodextrin (SBCD). The in vitro study showed enhanced amlodipine release and ocular permeation was seen in the HPMC complex with cyclodextrin and SBCD, both at a higher level. Results show enhanced permeation of amlodipine-HPMC film with sulpho butyl ether β-cyclodextrin [152]. Another bioavailability enhancement study was done by Morsi et al.; he studied the availability improvement and prolongation of ketorolac tromethamine for postoperative inflammation. In this, the gelling capacity pluronic 1 F-127 was 20% and pluronic1 F-127 14%/x HPMC K4M (14%). The mucoadhesive strength was increased by the addition of HPMC. The result shows sustained release, enhanced bioavailability, and prolonged residence time of the nano-dispersion of ketorolac tromethamine into the in situ gel for ocular delivery [153].

HPMC was studied for sustained release of ocular delivery. Wei et al. determined that in in vitro studies of 4% HPMC 606W in 15% P407 solution and 5% PEG4000 in 2% MC solution gives a gelation temperature and a sustained-release effect. In vivo studies showed a higher drug concentration in the cornea, aqueous humor, iris, and ciliary after 4 h to that of commercial BH suspension eye drops with Betaxolol hydrochloride (model drug). In poloxamer 407, methylcellulose is used as the carriers. A two times higher AUC and MRI of the in situ gelling eye drops was found comparable with the commercial product [132]. Ela et al. studied the HPMC to increase the permeation of an ocular antifungal drug (fluconazole) by using the anti-solvent precipitation non-ionization method. The stabilizers used were HPMC E3, xanthan gum, polyvinyl pyrrolidone K30 (PVP), Pluronic F-127 (PL F 127), Kollicoat IR (KL), and sodium lauryl sulfate (SLS). By using a goat cornea, ex vivo studies were done. The result of the ex vivo study shows improvement in treated fluconazole over untreated fluconazole. Optimization of both treated and untreated fluconazole was studied in rabbits. Particle size and zeta potential vary according to the type and pharmacokinetics parameter of the drug [154]. A similar study was performed by Gugleva et al., who worked on the thermosensitivity of situ gels for ocular delivery. The polymer used in their study was poloxamer 407 and by combining it with HPMC was made by the cold method and sol-gel transition. Gelling time and capacity were evaluated, and HPMC leads to a reduced phase-transition temperature. The gelation temperature (34 °C), pseudoplastic flow, and very good physical stability were found in doxycycline niosome in an in-situ gel form of 15% *w*/*w* poloxamer and 1.5% *w*/*w* HPMC. An in vitro study shows prolonged and sustained release of doxycycline than noisome alone. Results showed a significant therapeutic concentration and a sustained release of the drug [155]. In another study of an in-situ gelling system, the controlled release of latanoprost by the use of HPMC was studied by Khattab et al. An optimal factorial design was built of 4-factor concentrations of P127, P68, and HPMC, depending on the sol to gel transition temperature, the strength of the gel, and the mucoadhesive properties. The optimal formula showed a sol-gel transition of 34.3 °C, muco-adhesion of 0.06, gel strength of 23.13 g, a flux of 11.4 µg/cm^2^/h, even the anti-glaucoma effects rose 2.9fold and there was a reduction in the intraocular pressure (IOP) after 30 min and extended release to 8 h compared to conventional eye drops. This thermosensitive in situ gel of latanoprost is a good alternative to conventional drops [156].

HPMC was also used for drug recovery and to decrease the degradation of drugs. Terreni et al. studied antimicrobial peptide (hLF 1-11) derived from the N-terminus of lactoferrin, which is chemically, physically, and biologically unstable. In this study, the solid matrices containing mucoadhesive polymers were evaluated for rheology, hydration time, bio-adhesive property, drug content, and in vitro release of the formulation. The HPMC /T2/HA/hLF-11fd shows good drug recovery and chemical degradation was not seen for 6 months. This is a very promising result for pre-corneal delivery of anti-microbial peptides onto the ocular surface [157]. In another study, Maharana et al. showed that HPMC is used for the tear substitutes for dry eyes; in this study, they used CMC (0.5%), hydroxypropyl-guar, which contain polyethylene glycol 400 or propylene glycol, and HPMC (0.3%). In this, study they divided the patients into three groups: group-1 (CMC), group-2 (PEG/PG), and group-3 (HPMC). The result showed that HPMC and hydroxypropyl-guar containing PEG/PG was better than the CMC but HPMC improved more than PEG/PG [125]. Table 5 includes the comparison between the free drug and drug with cellulosic polymers and Table 6 indicates the marketed products of polymers.

**Table 3:** Application of HPMC in ocular drug delivery.

Cellulosic Polymer	Drug Used	Application of Polymer	Effect on Drug Property	Ref
HPMC	Fk506 (tacrolimus)	Improve bioavailability and efficacy, prolonged retention	Reduced surface tension of nanomicelles solution (33.61 ± 0.29 Mn/m) caused easy contact with ocular surface leading to improved retention and enhancing permeation and in-turn bioavailability	[150]
	Econazole	Increase the solubility and bioavailability	5 and 10% HPMC 15cp was preserved the supersaturation state of drug loaded SEDDS	[151]
	Ketorolac tromethamine	Sustain release, enhance bioavailability, and prolonged residence time	Enhanced AUC, Tmax and relative bioavailability that is 2.742 ± 0.11 µgh/mL, 11.57 ± 0.73 h and 250%	[153]
	Betaxolol hydrochloride	Prolonged release upto 85% in 6 h which is similar to higher concentration of P407 alone	Enhanced in AUC and MRT by 2 times compared to commercial preparation	[132]
	Fluconazole	Increase the permeation and stabilizer	Nanoparticle contaning drug was stabilized by 0.5 to 1% HPMC	[154]
	Doxycycline	Prolong and sustain release	Decreased burst release of drug	[155]
	Latanoprost	Controlled release upto 8 h	Anti-glucoma effect of in situ gel was 2.9 fold greater than eye drop	[156]
	Antimicrobial peptide (hLF 1-11)	Reduce chemical degradation	Mucoadhesive effect of HPMC improves the peptide interaction with ocular surface thus prevent rapid elimination of formulation	[157]
	HPMC and CMC	Tear substitutes	OSDI was significantly lower mean in HPMC containing group	[125]

**Table 4:** Clinical trials based on various cellulosic polymers used in ocular drug delivery.

Clinical Trial	Drug Used	Phase	Location of Work	NCT Number	Ref
HPMC 0.3% and sodium hyaluronate 0.18% for ocular surface disease in glaucoma	HPMC and sodium hyaluronate	Not applicable	Bangkok, Thailand	NCT01284439	[158]
Assess the safety and efficacy of CsA ophthalmic gel in subjects with moderate to severe dry eye disease	CsA ophthalmic gel, Hypromellose eye drop and placebo	3	China	NCT04541888	[159]
Effect of topical besifloxacin on ocular surface bacterial microbiota prior to cataract surgery	Besifloxacin, HPMC ophthalmic solution	1	Mexico	NCT04542759	[160]
An efficacy and safety study of bimatoprost alone compared with travoprost and timolol in glaucoma or ocular hypertension	Hypromellose (0.3%), Bimatoprost (0.01%), travatan (0.004%), timolol (0.5%)	4	Barrie, Ontario, Canada.	NCT01881126	[161]
Efficacy and safety study of bimatoprost alone compared with travoprost and timolol in glaucoma or ocular hypertension	Hypermellose (0.3%), Bimatoprost (0.01%), Travoprost (0.004%), Timolol (0.5%).	4	United state	NCT02097719	[162]
Evaluate the safety and efficacy of a new artificial tear for use after LASIK surgery	CMC and glycerin-based artificial tear	Not applicable	United state	NCT00544713	[163]
Safety and efficacy of Alphagan P and Lumigan in subjects previously treated with latanoprost for glaucoma and hypertension	Hypromellose (0.2%), Brimonidine tartrate (0.1%), bimatoprost (0.2%), latanoprost (0.005%).	4	United state	NCT01525173	[164]
Efficacy study of ketorolac and HPMC to treat dry eye	Ketorolac and HPMC	2	United state	NCT03693183	[165]
The effect of BAK on the blood-aqueous barrier of pseudophakic patients	HPMC and CMC	4	Brazil	NCT01280110	[166]
Efficacy, Tolerability, and comfort of Hypromellose eyedrop in patients undergoing LASIK surgery	Pre LASIK Hypromellose (0.3%), post LASIK Hypromellose (0.3%).	4	India	NCT00909324	[167]
Assessment of alcon’s ocular image quantification system	Olopatadine HCL, Patanol (0.1%), dextran 70 (0.1%), HPMC (0.3%)	4	United state	NCT01282138	[168]
Corneal protection used during cataract surgery	HPEC (2% gel), BSS	Not applicable	China	NCT02363530	[169]
Interval intraocular pressure in intravitreal injection study (IIII)	Hypromellose, travatan, timolol	Not applicable	Hong kong	NCT04868175	[170]

**Table 5:** Comparison between of Free form of Drug and Drug with Cellulosic Polymers.

Drug	Cellulosic Polymer Used	Effect of Drug in Free Form	Effect of Drug along with Cellulosic Polymer	Ref
Betaxol HCL	HPMC 606W, MC	No sustain release is seen, AUC and MRT is less and does not have prolonged drug release	Higher drug concentration after 4 h, AUC and MRT of in situ gel was 2 time higher than free drug, have prolonged drug release	[132]
Econazole nitrate	HPMC	Econazole under precipitation and have low aqueous solubility	HPMC sustain the supersaturated state by decreasing econazole precipitation, improve aqueous solubility of econazole.	[151]
Fluconazole	HPMC	NP is not stable compared to drug along with HPMC, particle size is larger less viscous formulation, permeation and pharmacokinetics parameter is less.	HPMC stabilized the NP formulation, increase polymer concentration reducing the particle size, and increase the viscosity of formulation Fluconazole NP enhance the corneal permeation and improve pharmacokinetics parameters.	[154]
Tacrolimus	HPMC	Permeation of drug is less, as it poorly water-soluble drug, therefore have less bioavailability and efficacy	Enhance penetration of hydrophobic drug, improve bioavailability and efficacy of the drug.	[150]
Olopatadine HCL	EC	Drug is inefficient because of low bioavailability,	Olopatadine doughnut CL along with EC shows sustained and extended release of drug with limited alteration to optical and swelling property of CL	[171]
Timolol maleate and metformin HCL	EC	It had low viscosity their rapid clearance from the eye, intravitreal is only option for delivery of drug to back of the eye so have more side effects and risk of infection and retinal detachment	Oleo-gel prepared from EC provide the viscosity to the formulation and give control or sustain release of drug which reduces the frequency of dosing, release of metformin form gelled emulsion is 1400 h and for timolol it is 2200 h that shows sustained release and drug loading is also increased.	[127]
Voriconazole	CMC	Drug have low residence time and bioavailability because of the rapid clearance of drug from the eye	In situ gel improves the residence time and bioavailability of the drug, formulation shows sustain release of drug	[172]

**Table 6:** Marketed products based on cellulosic polymers for eyes.

Drug	Name of Marketed Products	Dosage Form	Polymer
HPMC	RETAINE HPMC-hypromellose 2910	Solution/drops	HPMC
HPMC	IO Gel (HPMC solution)	Solution	HPMC
HPMC	OCCUGEL 2%	Solution	HPMC
HPMC	VIBKOOL	Solution	HPMC
HPMC	Lubricate	Solution	HPMC
HPMC	Eyemist	Eye drops	HPMC
HPMC	IRIMIST	Solution	HPMC
HPMC	BLINK lubricant	Eye drops	HPMC
HPMC	MEDIVISC forte	Eye drops	HPMC
HPMC	Tobmat	Eye drops	HPMC
Flurbiprofen and HPMC	Flumat	Eye drops	HPMC
Sodium CMC	A-CMC	Eye drops	CMC
Calcium CMC	CELLU TEARS gel	Eye drops	CMC
Naphazoline HCL, Camphor, Menthol and chlorpheniramine maleate	BRISCOOL	Eye drops	CMC
Sodium CMC 0.5%	LUBRIZETHIC	Eye Drops	CMC

#### 10.1.3. Carboxymethyl Cellulose

Carboxymethyl cellulose (CMC) is a biocompatible, biodegradable, non-toxic, and water-soluble ether derivative of cellulose; therefore, it is used for various purposes (Table 7). CMC is white to cream-colored, odorless, tasteless, free-flowing powder, and sodium CMC is generally called CMC [173]. It is an anionic, linear anhydrous-glucose polysaccharide, water-soluble, and generally recognized as safe (GRAS) [174,175]. By β-1,4-glycosidic bonds the polysaccharide repeating units are connected. CMC differs from cellulose as CMC has anionic carboxymethyl groups (CH_2_COOH), which are replaced by the hydrogen atom of a hydroxyl group of cellulose and it was first synthesized in 1918 but the commercial preparation was developed in 1920 in Germany [176]. Cellulose precursors are corn cob [177], corn stalk [178], corn husk [179], durian rind [180], maize stalks, pineapple peel [181], orange peels, sugarcane bagasse [182], asparagus stalk [183], and other materials such as waste paper [184], waste textile [185], knitted rag [186], cotton gin waste [187], waste cotton linter, etc. Apart from pharmaceutical use, it is also used in detergents, food, paper industries, oil drilling mud, and as a hydrogel in delivery systems and so on. There are many patents related to cellulosic polymers (Table 8). CMC can absorb a large amount of water and swells, and when it swells, the drug diffuses out from the hydrated layer of the swollen mass and shows its effects. Hence, its forms a metallo-polymeric material by chelating with the metal ions [188].

Neslihan et al. studied the improved residence time and bioavailability of the in situ ocular gel formulation of Voriconazole, used in fungal keratitis treatment. To prepare the thermosensitive in situ ocular gel, poloxamer 407, poloxamer 188, and CMC were used. The formulation prepared by use of this material showed a gelation temperature of 29–34 °C. They were evaluated for sterility, antifungal activity, stability, in vitro drug release, in vivo studies, and ex vivo studies for penetration and permeation. All three formulations showed good sustainability of drug release. This showed a good effect of CMC for increasing the residence time and bioavailability [172]. Similarly, Hassan et al. studied the enhancement of bioavailability of an in-situ gel of voriconazole for ocular insert loaded with a noisome suspension. In this study, noisome was made with span 40 and span 60 with pluronic L64 and pluronic F127. Then, the noisome was evaluated for entrapment efficacy, poly-dispersity index, mean vesicle size, zeta potential, and in vitro drug release. An in situ gel was made by sodium CMC and sodium alginate and then evaluated for surface morphology, surface pH, water uptake, mucoadhesive properties, and in vitro release; this CMC sustains the release of the drug and prolong its effect [189]. In another study, sustained release of a drug by the use of CMC was studied by Sarimsakov et al. In this study, he used the soluble antiviral eye films of a polymeric form. The materials used were aqueous-soluble sodium CMC and derivative of sodium CMC that contains a chemically bound natural polyphenol-gossypol with the quantity of polymerization of 630 ± 20 and 0 mole%, where soluble CMC has a degree of substitution of 0.85 ± 2. CelAgrip is the antiviral drug substance, and sodium CMC-CelAgrip showed the prolonged effect of the drug, and the film is transparent and non-irritating [190]. Another study is about the bioavailability enhancement and prolonging the residence time of beclomethasone dipropionate (BDP), done by Gaballa et al. In this study, glyceryl monooleate (GM) Cubosome was made, and the stabilizer used was poloxamer 407 and Solulan C24. The particle size of Cubosome was 100–278 nm, the EE% was 94%, and they found significant trans-corneal permeability (*p* < 0.05). Then, the optimized Cubosome was incorporated into the CMC gel to form a Cubo-gel; this gel shows better rheology, enhance ocular tolerance, and superior anti-inflammatory activity compared to a suspension of BMD. The cumulative % of drug release from the Cubosome and Cubo-gel is 18.7% and 29.7%, showing an 8.64- and 13.6-fold increase in release compared to the control suspension BDP (2.17%). Increasing the concentration of CMC from 0.5 to 1% causes an increase in viscosity and decrease in drug release, leading to a decrease in the cumulative % release and had a prolonged residence time [191].

Jiang et al. studied CMC combined with Mistletoe for dry eyes in postmenopausal women. Here, one group was given the eye drop whereas another group was given normal saline eye drops. Patients were diagnosed with diastolic pressure, systolic pressure, glutamic-pyruvic transaminase, glutamic oxaloacetic transaminase, and urine creatinine for safety and efficacy of the treatment after eight weeks. Ocular surface, OSDI, tear protein, and tear film functionality were evaluated after two months of treatment; this shows a slight change for normal saline whereas enhancing the effect in CMC combined with the mistletoe eye drop group. The result shows mistletoe combined with CMC improves the symptoms of dry eye [192]. In another study, Prasad et al. studied the safety and efficacy of CMC (0.5%) and HPMC (0.3%) for dry eyes due to computer vision syndrome. Efficacy parameters checked were the ocular surface disease index (OSDI) score, tear film break-up time, and Schirmer l test score, and the safety were checked in all. OSDI was reduced in both, the tear film breakup time was enhanced in both, and the Schirmer l test was increased in both, showing CMC and HPMC are both effective and safe for dry eye occurrence due to computer vision syndrome (CVS) [193]. Titiyal et al. studied the function of topical chloroquine as a topical lubricant for dry eye disease. In this study, CMC 0.5% and chloroquine with CMC 0.5% were evaluated for 3 months. Results show the OSDI score was better in chloroquine with CMC than CMC alone; the Nelson’s score for the CMC group was 1.60 ± 0.77 and for chloroquine CMC 0.92 ± 0.69, and Schirmer test and OSDI were better for chloroquine with CMC, showing this is effective for dry eye disease [194]. A similar study based on treatments of dry eye was studied by Lievens et al.; in his study, he compared two lubricant artificial tear formulations, which have more viscosity. He used CMC 1.0% with glycerin 0.9% that contain an osmo-protectant compared with CMC 1.0%. The CMC with glycerin at 7 days shows significant enhancement in a baseline of OSDI (all patients *p* < 0.001, severe *p* < 0.001), TBUT (*p* < 0.001), and corneal stain (*p* = 0.031), and other results are the same I for both formulations. Adverse effects reported were blurred vision. This showed CMC GLY formulations are the same as CMC alone; therefore, they both were better for dry eye disease [195].

The CMC was combined with hyaluronic acid to get synergistic effects. In one of the studies, Aragona et al. combined the two polymers CMC and HA and compared this with CMC alone to treat dry eye. The primary evaluation parameters OSDI and secondary are TBUT, surface stain, Schirmer test, and visual analog scale scores in dry eye patients. The safety parameters are adverse events, bio-microscopy, and visual acuity. This study shows the OSDI score at 90 days for CMC-HA and CMC are −16.9 ± 17.5 and −16.0 ± 16.1, and the results show CMC-HA was as effective as CMC for dry eye disease. Both formulations have significantly enhanced properties of OSDI, TBUT, and surface stain for a dry eye [196]. Another study of polymer combination was done by Simmons et al. In this study, they used a water-soluble polymer, which helps to improve the residence time, moisture retention, and mucin binding, and increase the corneal healing. In the study, CMC and HA alone and the combination was prepared and checked for viscosity rheometry, where the viscosity test showed the enhanced shear rate and simulating eye movement. The viscosity of CMC 0.5% and HA 0.1% was 2.5 and 5.7 cp whereas the viscosity of the combined solution was 13.1 cp, which is 60% higher. The results show that the combination of CMC and HA has a synergistic effect in low shear viscosity and high shear viscoelasticity; these data tells us that the CMC–HA combination can be used for dry eye as artificial tears, which has less blurred vision and stickiness when blinking [197].

In another study, Sodeinde et al. studied the encapsulation efficacy of CMC of vitamin A in an oil phase; he modified the native cellulose to CMC and cellulose acetate by etherification or acetylation and then studied the material for structural crystallinity, morphology, thermal studies, chemical composition, degree of substitution, and acetyl modification. This modification results in decreased structural crystallinity, and an enhanced amorphous nature when scanning in a wide X-ray diffractometry. This shows CMC enhances the stability of a vegetable oil–water emulsion and significant encapsulation of vitamin A [198]. In another study, Downie et al. studied the nano emulsion artificial tear efficacy by use of CMC and glycerin, flaxseed oil and castor oil, and levocarnitine, erythritol, and trehalose as an osmo-protectant were compared with the artificial tear that contains the same ingredients, except for trehalose and flaxseed oil. A 7-day run-in period of topical CMC (0.5%) was taken, showing both emulsified artificial tears have an improved baseline in the ocular surface disease index (OSDI) score, ocular surface staining, and tear film breakup time (TBUT) [199].

#### 10.1.4. Ethyl Cellulose

Ethyl cellulose is a linear, non-toxic, non-swellable polysaccharide [211] derivative of cellulose [149], it is insoluble in water and soluble in organic solvents, and has great mechanical properties [212]. It can be produced by Williamson etherification of cellulose and ethyl chloride. It is mainly used for thin-film coating, acts as a binder taste masking agent, and modified release excipient [213]. It can be modified at the C2 and C6 positions with 2.1–2.6 of the DS range. The reactivity of ethyl cellulose is low for more functionalization because the remaining free hydroxyl groups are present on C2 and C3. Ethyl cellulose has various important properties, such as chemical stability, thermo-plasticity, and avoid alkali and salts degradation, and even low water absorption capacity; therefore, it has application in paints, lacquers, varnishes, and is also used in hair sprays. It has very good solubility in organic solvents that make it a good candidate for various formulations [214]. Ethyl cellulose is biocompatible and has a self-assembling capacity; therefore, it is widely used in biotechnology [215].

Obiedallah et al. studied the improved therapeutic effect and decrease in the systemic adverse effect of acetazolamide, formulated as microsponge in situ gel for ocular delivery. Microsponges were prepared by using various proportions of ethyl cellulose. Further, the developed microsponges were incorporated into the Pluronic F-127 (25%) in situ gel and was compared with a free drug gel formulation. Drug and polymer in ratio 2:1 have shown a high entrapment efficiency (82%) with a particle size 10 µm and polydispersity index of 0.22; these results best suited for ocular delivery. [216]. Zhu et al. studied the controlled release of diclofenac sodium from the developed by contact lenses. However, this system had some disadvantages related to storage stability, drug loading, and limited release duration. To overcome these limitations, the research group embedded an inner layer of contact lenses, which showed pH triggered extended release of the drug. The inner layer was made by a blend film of ethyl cellulose and Eudragit S 100 and the outer layer was made by p HEMA hydrogel. An in vivo study showed sustained release for 24 h in the tear film, which revealed enhanced corneal resistance time; therefore, this embedded inner layer by ethyl cellulose and Eudragit can be used for extended or control release [217]. A similar study based on controlled release of drugs was done by Maulvi et al. In this study, drug control release was done in contact lenses, but the optical and physical properties of the lenses changed the drug loading. Timolol maleate (TM) was loaded in an ethyl cellulose nanoparticulate-laden ring in a hydrogel contact lens; this hydrogel contributed to the controlled release without altering the characteristics of the contact lenses. Lenses were prepared by dispersing the TM encapsulated ethyl cellulose nanoparticles in acrylate hydrogel. In vitro studies showed sustained release of the drug for 168 h and the drug loading was 150 µg. In vivo studies showed significant enhancement in mean residence time and AUC and also shows the decrease in intraocular pressure for 192 h, which was compared to the eye drops of TM [218] (Table 9). Another similar study related to timolol maleate loaded with ocusert was studied by Nair et al. In this study, ocusert was made with sodium alginate (hydrophilic polymer) and ethyl cellulose (hydrophobic polymer), and polyethylene glycol as a plasticizer. The ethyl cellulose concentration used was 1–6%. The formulation was evaluated for drug entrapment efficacy (94–98%) and content uniformity (93.1 ± 0.264–98.0 ± 0.321%), and the in vitro drug release shows (83.42 ± 0.35%) after 12 h and the ex vivo study gave a significant result and a decrease in intraocular pressure was found after 3 days. An increase in the concentration of ethyl cellulose decreased the permeability coefficient of the timolol maleate in the ocular insert. Therefore, for sustained release of drug from the ocusert, a high ratio of ethyl cellulose is needed and this was compared with the marketed eye drops of timolol. Result shows sustained or delayed-release from ocusert through a corneal membrane [219]. Another study of controlled release of the drug was done by Balzus et al. In this study, dexamethasone-loaded ethyl cellulose, Eudragit RS, and a combination of both EC and Eudragit RS NP was prepared. The formulation was evaluated for drug release, drug penetration, and NP toxicity. The study shows that ethyl cellulose NP (1.4–2.2%), which is larger in size and has a negative zeta potential because of adsorption of the hydroxyl group, has a better loading capacity than Eudragit (0.3–0.7%), which is smaller and have a positive zeta potential because of the quaternary ammonium group in the Eudragit RS surface; this positive charge decreases the NP–NP aggregation and it has a lower viscosity than ethyl cellulose. The polymer combination in a ratio of 1:1 showed a smaller particle size (105 µm), positive charge NP (+37 mV) with drug loading (1.3%). As the drug–polymer ratio was decreased, there was a decrease in the drug release and drug penetration. However, when the Eudragit and ethyl cellulose blend was used, the drug release and drug penetration was increased [220].

#### 10.1.5. Hydroxyethyl Cellulose

Hydroxyethyl cellulose is a water-soluble cellulose ether; it is produced commercially by a chemical reaction between ethylene oxide and cellulose. Hydroxyethyl has randomly substituted glucopyranose units at positions 2,3,6 by hydroxyethyl groups and the side chain will be mono, di, or trimer. It is a biocompatible, biodegradable, hydrophilic, non-ionic water-soluble, low toxic, and non-immunogenic cellulose derivative [128] (Table 10). HEC can be used as a stabilizer, thickener, or coating material, and because of the high aqueous solubility, HEC can be used in various applications that as film-forming materials, in pharmaceuticals, in cosmetics, biotechnology, and in ophthalmic preparations [221,222,223]. 

France et al. studied the sustained release of dorzolamide (hydrophilic drug) as an anti-glaucomic agent that is an ocular insert that can decrease the dose frequency, low systemic exposure, decrease the adverse effect, and improve patient compliance. They used chitosan and HEC ocular inserts to produce sustained release of the drug. The inserts were tested for effectiveness in a rat model and in vitro release shows that at 3 h, 75% of the drug was released from the inserts, whereas ex vivo studies show that the biodistribution of 99 m Tc dorzolamide is more than 50% 18 h after administration. The thickness of the inserts was 40 to 70 µm. Dorzolamide is dispersed in the polymeric matrix which causes sustained release of dorzolamide. This system is a control–release system that has an advantage over conventional ones, as inserts allow the sustained release of drugs (Table 6); this leads to a decrease in the dosing frequency and increase in patient compliance [68].

Mohammadi et al. studied the improvement in the delivery of Ketorolac tromethamine; in this study, they used Eudragit L-100 NPs with ketorolac, which was incorporated into the PVA and hydroxyethyl cellulose (HEC) films. The encapsulation efficacy and physicochemical property and physiochemical parameters, such as % moisture absorption, % moisture loss, thickness, and folding endurance, were evaluated. Results showed a mean particle size (153.8–217 nm) and zeta potential −10.8 to −40.7 mV, and the loading of the drug increases as the quantity of Eudragit and HEC increases, and the thickness of the inserts was 0.072 ± 0.0098 to 0.0865 ± 0.0035 mm. The thickness and folding endurance of the inserts increased with an amount of polymer; therefore, Eudragit NPs loaded with the PVA and HEC is an effective carrier for delivery of the drug. In this study, the inserts were formulated to reduce the frequency of ketorolac and enhance patient compliance [224]. A similar study to improve the drug delivery was done by Taghe et al. In this study, azithromycin-loaded Eudragit L 100 NPs inserts, which are sustained release and have increased ophthalmic performance, were prepared by use of HEC, HPMC, and a plasticizer such as PVA. The optimized formulation has a particle size of 78.06 ± 2.3 nm, zeta potential around −2.45 ± 0.69 mV, polydispersity index of 0.179 ± 0.007, and EE of 62.167 ± 0.07%. The thickness of AZM-HEC was 0.156 ± 0.008 mm and AZM-HEC was 0.164 ± 0.005 mm, which is suitable for insertion into a cul-de-sac and show no irritation of the eye. The tensile strength and elongation at the break were higher in HEC films compared to HPMC film and the pH was between 6.66 ± 0.05 and 6.83 ± 0.055, which is near to the ocular pH 6–7.6; this shows that these inserts can be applied to the ocular tissue with no irritation [225]. Allam et al. studied the Betaxolol noisome that is a pH-responsive in situ gel for the prolongation of pre-corneal retention of Betaxolol. Optimal nano-dispersion was incorporated into the pH-responsive in situ gel by use of HEC and Carbopol 934P. The prepared gel was translucent, pseudoplastic, mucoadhesive, and had sustained release. The formulation was evaluated for vesicle size, morphology, size distribution, surface charge, and EE. This formulation had to extend the decrease in intraocular pressure and enhanced relative bioavailability was seen compared to the marketed one. Therefore, niosome may be an effective carrier for the ocular drug delivery [226]. A similar study of sustaining the release of the drug was done by Destruel et al. In this study, a mucoadhesive and ion active in situ gel of Phenylephrine and Tropicamide was prepared by use of hydroxyethyl cellulose and gellan gum. The suitable property of ocular administration was checked by the physicochemical characteristics and rheological properties such as viscosity, and the gelation ability was checked. A new rheological technique was established to check gel resistance in simulated eye blinking. Fluorescence intensity determined the extended residence time on the surface of the eye compared to the conventional eye drops, which showed an extended-release of the drug from this oxidized hydroxyethyl cellulose hydrogel [227]. In another study, Pennington et al. studied infectious ocular disease; in this study, povidone-iodine (0.5%) alone or in combination with HEC (1%) was evaluated for its efficacy. Povidone alone is effective for feline herpesvirus type 1, Chlamydia felis, and Mycoplasma felis, whereas povidone with HEC has an additive effect for feline herpesvirus type1 and C. felis. This additive effect may be due to an increase in culture medium viscosity because of the HEC [228]. A similar study about ocular disease was done by Mckenzie et al. In this study, cysteamine is used to treat corneal crystal deposition. The pre-formulation evaluation of the cysteamine-containing gel was evaluated, and the suitability of the drug was identified by analysis of the rheology, bio-adhesion, dissolution, and stability. Various polymers were checked for the above properties and as a result, the three polymers that suit ocular delivery were hydroxyethyl cellulose, sodium hyaluronate, and carbomer 934. As sodium hyaluronate and HEC are four to five times more viscous, carbomer is the clear solution [229]. Kang et al. prepared a novel oxidized hydroxyethyl cellulose and allyl co-polymer-based hydrogel. In this study, they used a hydroxyethyl cellulose molecule chain as the biomacromolecule template, from which the Schiff base and mechanical properties were obtained by borate and hydrogen bonds, which have fast recovery with little or no hysteresis and have significant compressive capacity. In oxidized hydroxyethyl cellulose, a hydrogel functionalized allyl spiro-oxazine derivative was applied to endow photo- and pH-sensitive substrates. This hydrogel has a good pH environment adaptability and was found non-toxic in the in vitro test; this oxidized hydroxyethyl cellulose hydrogel had significant use as a safe development, is fashionable, such as for pH-detectable contact lenses [63].

#### 10.1.6. Hydroxypropyl Cellulose

Hydroxypropyl cellulose is a water-soluble cellulose ether, on the cellulose backbone the hydroxy group, and is propylated to produce hydroxypropyl cellulose [230]. On a commercial basis, it is prepared by reacting propylene oxide with alkali cellulose at a high pressure and temperature to get a substituted derivative of the cellulose ether. Due to the high concentration of hydroxypropyl, 70%, it is highly plastic and hydrophobic compared to other cellulose ether derivatives. It has a temperature-dependent aqueous solubility and is also soluble in polar organic solvents such as ethanol, methanol, isopropyl alcohol, and acetone. Soluble at below the cloud point temperature, 45 °C, it is commercially available in various viscosity grades, and has a molecular weight of 20–1500 kDa [231,232]. The low grade is used as a binder whereas the coarse grade is used in the solution formulation for a lump-free solution [233].

Moosa et al. studied the topical ocular delivery of instantly soluble solid eye drops (ISED) in which the model drug used was timolol maleate. Materials used here were hydroxypropyl cellulose and Pluronic F68 as the matrix-forming agents and the instantly soluble solid eye drops were lyophilized, and the porous nature of the ISED showed rapid fluid ingression, immediate hydration, and dissolution of the ocular matrix. Solubility is increased by the use of polyacrylic acid and the anti-collapsing agent used was di-glycerin, and the matrix resilience was maltodextrin. It was evaluated and optimized for texture, disintegration, and a mean dissolution time of ISED. The result showed that ISED has fast disintegration of 0.20 s and drug release was 79–96% and enhances corneal drug permeation. Both polymer Pluronic F68 and hydroxypropyl cellulose were water-soluble, which absorb the fluid and increase the residence time of the drug as compared to the conventional eye drops [129].

### 10.2. Cellulose Ester Derivatives

#### 10.2.1. Cellulose Acetate

Cellulose acetate is a semisynthetic ester derivative of cellulose, on the cellulose backbone hydroxyl group, and was substituted with the acetyl group [234,235]. Commercial cellulose acetate (CA) was prepared by esterification of cellulose with acetic acid and the degree of substitution [236] changes the physical properties of the cellulose acetate, such as aqueous solubility and viscosity. The high degree of substitution means low water solubility and 0.5–1.1 DS gives water solubility. There are two types of cellulose: water-soluble cellulose acetate (WSCA) and insoluble cellulose acetate (ISCA). The degree of substitution by the acetyl groups for WSCA was 0.78 and ISCA was 2.4, and the degree of polymerization for WSCA and ISCA was 124 and 301. Cellulose acetate has various applications, such as in the textile and pharmaceutical industries [237]. Cellulose acetate is a non-toxic, non-irritant, and biodegradable polymer; it is heat resistant and less hygroscopic. Partially acetylated cellulose acetate is in the range of 29–44.8%, which is mono, di, and triacetate [238].

Hongbo Cheng et al. studied the sustain release of Betaxolol hydrochloride, which is a water-soluble drug by preparing it in an inner layer embedded in a contact lens; the materials used were cellulose acetate and Eudragit S100 for preparing the inner layer, whereas silicone hydrogel was used for the outer layer. These were evaluated and optimized for a polymer ratio in the blend, drug release in vitro, and also evaluated for drug-polymer interaction, erosion, and swelling of the polymers. The stability study was also taken to check the drug-release mechanism. Result of the in vivo study in rabbits showed a sustained release action of the contact lenses for 240 h in tear fluid, which determined the prolonged residence time of drug release. Drug release was 25% in STF (pH-8) in the first 3 h and 66% drug release from 72 h, so cellulose acetate is effective in extending the drug release with less burst release. This study determined that cellulose acetate can sustain the release of drugs from the film into the eyes but at pH 6.8 fast drug-release was seen, which indicates a premature drug release during production or at shelf, if in this study cellulose acetate is combined with the Eudragit S100. Hence, CA/Eudragit S100 is a promising approach for sustaining drug release [130]. Applications of HPC, CA, and CAP are shown in Table 11.

#### 10.2.2. Cellulose Acetate Phthalate

Cellulose acetate phthalate is a derivative of cellulose ester. The common name of CAP is cellacefate, cellulose acetate, cellulose ester, and a patented aqueous enteric coating called Aquateric, which are latex particles, re-constituent colloidal dispersion developed by the FMC corporation; this is the semisolid and solid spherical polymer of the CAP and pseudo-latex such as Aquacoat CPD by FMC. It is as a natural polymer that acts by dissolving at pH > 6; it is hygroscopic and therefore susceptible to moisture. CAP is used as a control release, enteric coating for tablets and capsules, for delayed-release [239,240]. CAP latex is the choice of polymer because of compatibility with drug molecules and can convert from sol to gel at pH 4.2 to 7.2, which is the phase transition that is triggered based upon pH, and is stable at a low pH, and thus can be used as an in-situ gelling vehicle. CAP pseudo-latex 30% by weight shows a high increase in viscosity compared to the change in pH. CAP latex is a non-irritant to the eye, but the discomfort can be because of coagulation of the solution in a cul-de-sac. The evaluation of the precorneal residence time of CAP latex was done in a γ-scintigraphy study and that shows the CAP latex have superiority to other solutions, having the ability to prolong the pilocarpine corneal residence time, shown by in vitro scanning electron microscopy, as well as in vivo; by incorporating methylene blue in rabbits, the gelation capacity of the CAP latex was determined in ophthalmic preparations. By measuring the pharmacological response efficacy and by γ-scintigraphy, the precorneal residence time of the pseudo-latex formulations was assessed [241,242]. Subramanian et al. studied the dual stimuli-responsive system that contains ibuprofen-loaded CAP that is pH-responsive nanoparticles that are dispersed in pluronic F127 and F68, which is a temperature-responsive gel-forming solution. Pluronic solution F127, 21% and F68, 10% NP at ocular temperature have a good phase-transition behavior and moderate viscosity, which is preferred for ocular delivery. An NP size is about 159 to 356 nm, and the in vitro drug release was 70% at 8 h, and the prolonged retention time and control permeation of the drug was 180 for 2 h, which is responsive to pH and temperature [105].

### 10.3. Nanocellulose

Nanomaterial has a size range of ≤100 nm, which may be in form of particles, tubes, fiber, and rods, and they are applied widely. Nanomaterial addition may cause pore refinement, increased durability, and strength, which is because of the multiphase, complex, and nanostructured cementitious composite [243]. Cellulosic nanomaterial is beneficial due to its physicochemical properties, such as high stiffness, strength, and surface area, and also they have a good interaction with water, inorganic, and polymeric substances [244]. Nanocellulose enhances the reactivity and contact between the constituents and forms the highly functional and performance-graded material [245]. Nanocellulose is an organic material, and therefore is obtained in many forms such as cellulose filaments and cellulose nanocrystals. Microcrystalline cellulose is the crystalline form and the cellulose microfibrils and cellulose nanofibrils are the fibrillated form of cellulose. Microbial cellulose or bacterial cellulose nanocellulose is synthesized from bacteria, and bacterial cellulose exhibits high purity, strength, and water-retention properties [110]. Nanocellulose is divided into three types, such as bacterial NC or bio-cellulose or microbial cellulose, cellulose nanocrystals or nanocrystalline cellulose, and cellulose nanofibrils or nano or micro fibrillated cellulose [244,246]. Nanocellulose is widely used in ophthalmic applications, as incorporated into the hydrogel.

Traditionally, the polymers used for hydrogel-based ocular delivery are poly(vinylpyrrolidone), poly (methyl methacrylate), poly (hydroxyethyl methacrylate), cellulose acetate, and silicon materials. For ocular applications, some important factors are considered, such as biocompatibility, mechanical stability, optical property, and oxygen permeability, also the materials used should not have any immunogenicity or toxicity. Hydrogel has a high water content, thus they are comfortable and have good lubricity, but hydrogel-based soft contact lenses are mechanically weak because of a high water content; therefore, nanocellulose is incorporated to overcome the drawback of plain hydrogel-based soft contact lenses [247].

Tummala et al. studied the TEMPO-CNC PVA hydrogel for ocular use where PVA is used to enhance the viscosity of eye drops and internal lubricant for contact lenses. Earlier for corneal implants, bacterial cellulose with PVA was used with a low optical transparency, 75% at 610 nm. CNC-PVA hydrogel contact lenses have mechanical properties similar to that of a rubber. It has good elasticity, softness, 95% transparent in a visible light range, and moderate ultra-visible property. It can retain its convex shape as it contains 90% water. Due to the similarity in the interface between CNC and PVA, the refractive index, width, and thickness of the CNC of the contact lenses are transparent [248].

Tummala et al. prepared the CNC PVA hydrogel that has a contact angle of 12°, which is low; a high oxygen permeability of 66 A° ×10–11 Dk; and a similar refractive index of distilled water (1.33), which is comparable to the marketed contact lenses. These lenses have a high elastic modulus with a Gl of 16 kPa. Hydrogel lenses are ductile in nature and the stress–strain curve of CNC PVA lenses is hyper-elastic in nature and has a failure strength of 0.15 MPa [249]. The gel can stretch 300% in size compared to the original size. These lenses can avoid 12 interrupted sutures without any tears. With corneal epithelial cells, cytocompatibility is tested. Cell growth is faster due to the hydrogel stimulating the growth of cells on a surface of hydrogel in three days. These showed it is a promising implant material, and strain-induced stiffening is seen in hybrid gels [250].

### 10.4. Bacterial Nanocellulose

Bacterial nanocellulose is a natural polysaccharide that is secreted by non-pathogenic bacterial strains. Bacterial cellulose can easily be modified to increase the function and properties of BC and various nanocomposites are developed [251,252]. It can be obtained as water-insoluble hydrogen in the interface between liquid and air from Komagataeibacter xylinus cultures [253]. BNC hydrogel is free of endotoxins after cleaning and contains nanocellulose fibers they are oriented in the same manner as collagen. Bacterial NC is porous, have a high water-containing capacity, and have a high surface area; because of these properties, BNC is used for biomedical application [254]. BNC is applied for skin tissue regeneration and has marketed products such as epicyte (jenacell); it is also used as a coating material for implantable devices such as a Hylomate pouch as an anti-fibrotic agent [255].

Ocular surface disorders affect the cornea, vision disturbance, and quality of life, such as corneal trauma, infectious ulceration, and burns. Conjunctival flap operations are used for these cases, but it causes postoperative visual loss. To overcome these, human amniotic membrane patches are used due to their regenerative effects. The human AM has two different sides, such as an epithelial side and spongy stromal side, which is made of cuboidal cells and later with collagen fibers, and this is placed in the corneal damage area but degrades rapidly before the healing time; it has a short shelf life and is costly. To overcome this, bacterial nanocellulose is used. Micheal et al. studied corneal trauma and ulceration, which cause blindness. For this amniotic, membrane graft is used as a treatment for blindness but a major limitation is its availability, high cost, and short shelf life [256]. BNC has very good properties for a corneal bandage that is highly liquid, has holing ability, flexibility, biocompatibility [257], and not of animal origin. Testing of BNC for its suitability as a corneal bandage, BNC has high mechanical resistance for sutures and is stable, which was checked by in vitro and ex vivo conditions other than amniotic membranes. Bacterial nanocellulose offers a good shape of the ocular globe and can be easily modified as it is heat sterilized and stable for a long time, and therefore can be used as a surface bandage [258].

### 10.5. Cellulose Nanocrystals

CNC belongs to a class of nanocellulose, and also called cellulose nano-particles or nanocrystalline cellulose (NCC) or cellulose nanowhiskers. It is a sustainable biomaterial, and have a unique structure and physiochemical properties, such as low density, biocompatibility, biodegradability, renewability, adaptable surface chemistry, non-toxic, good mechanical properties, and optically transparent [259]. It is used in wide fields such as pharmaceuticals, electronics, biomedical, nanocomposites, membrane super-capacitors [260,261,262] high crystallinity, and mechanical properties [263]; it also has other properties, such as being a co-stabilizer, and act as enhancers for covalent attachment of drugs; hence, it is also used in biomedical products [264]. CNC is extracted from the acid hydrolysis of cellulose, and the steps involved are acid hydrolysis under control conditions. By addition of water, interruption of the hydrolytic reactions, to remove acid extensive dialysis, is done. For dispersion of particles, mechanical treatment by sonication is done, and uniform and stable suspension is obtained; after that it is dried when the solid CNC is required [265]. These are needle-shaped materials that get fitted into the ocular microfibrils when their amorphous sites get destroyed [266].

Vakili et al. developed a locally delivered mucoadhesive hydrogel with superior activity in cisplatin (CDDP) for colorectal cancer by grafting the poly (acrylic acid) onto cellulose nanocrystals, prepared as a hardcore bottle-brush polymer at a ratio of 6, 9, and 12 of CNN: PAA *w*/*w*; this was evaluated by acid–base titrations, FTIR, mucorheological, electron microscopy, and in vitro drug release against human HCT-116 colorectal cancer cells. CNC PAA 9 has superior rheological behavior compared to CNC and gels. The CNC PAA 9: CDDP complex has significant enhancement in the IC50 of a drug that has a 3-fold increase against HCT 116 cells. CNC PAA does not have any cytotoxicity, and these results have shown that CNC PAA is a good method for local platinum delivery in colorectal cancer as a mucoadhesive hydrogel [267]. A similar study based on CNC was done by Sarkar et al., the main goal of the study being to get good bioavailability and a prolonged residence time. They studied the behavior of CNC during in vitro release of pilocarpine HCL and during the in-situ gelation of poloxamer. An enhancement in gel strength was seen due to the hydrogen bonding in the in-situ gel, and it also has sustained release of the drug through the gel compared to the poloxamer gel, and the drug was released based on Fick diffusion. This shows that CNC has a very good gelation property, gel strength, and dry release through it [112]. CNC is also used for the hydrophilic polymer matrix as a reinforcing agent, and the modified CNC is used for the hydrophobic polymers [259]. It is also used in electrospun to form nanofibers [268,269]. 

### 10.6. Cellulose Nanofibers

Cellulose nanofibers are abundant in nature, biodegradable, renewable, high strength, and cheaper, with very good properties as they are nano in size. Isolation of CNF is from various cellulose sources but a separation of the cellulose nanofibers is the major challenge [270]. CNF has a high surface area, high elasticity, rigidity, and unique optical characteristics [174,271]. CNF is prepared from the cellulose sludge; first, the cellulose sludge is mixed with distilled water, and then it is diffused to 3% concentration with a mechanical blender at a speed of 3000 rpm for 10 min, and then this suspension is passed through a mechanical grinder until a gel is obtained [266]. CNF is 2 to 20 nm in diameter and few micron meters in length, and it has a crystalline and amorphous site [272,273]. They are prepared from cellulose microfibers and developed by mechanical fibrillation, which involves micro-fluidization, homogenization, and ultrafine grinding of the cellulose molecules based on the source of the cellulose, and the pre-treatment, such as with chemical, mechanical, or enzymatic pre-treatments, which decreases the energy input and enhances the quality [266]. To improve the mechanical properties and environmental impact, there is a need for surface modification of the CNF, and therefore pre-treatment is required to enhance the interaction between the matrix and fiber, to increase the nanocellulose fiber’s moisture absorption barrier properties, and to increase the roughness and hydrophobicity of the surface [274]. Various methods of surface modifications are the adsorption method, and the chemical methods include silylation, acetylation, or esterification, carbonylation, and functionalized CNF reactions, modifications by polymer grafting, and bacterial modifications [246].

## 11. Safety and Toxicity of Cellulose in Ocular Drug Delivery

As the ocular tissue is extremely sensitive, it is essential to establish the safety and toxicity of the ophthalmic formulations. Ocular toxicity includes inflammation and atrophy of the optic nerve and inner retina, loss of white matter, and gliosis of the occipital and parietal lobes, causing various degrees of blindness [275]. Various toxicity studies, such as Draize and low volume eye tests, and non-ocular models, such as hen’s egg test-chorioallantoic membrane (HET-CAM), have been developed to assess the safety of the formulations. Organ/organotypic methods, such as chicken enucleated eye test, and cytotoxicity assessments, such as 3-(4,5-dimethylthiazol-2-yl)-2,5-diphenyltetrazolium bromide (MTT) assays, lactate dehydrogenase leakage, and fluorescein leakage, also help in assessing the toxicity of the developed formulations [276]. Orasugh et al. evaluated the toxicity of an in-situ nanocomposite gel by hemolysis and lactate dehydrogenase assay. The study showed that the in-situ gel loaded with cellulose nanocrystals (CNC) was hemocompatible when incubated in blood samples of healthy human volunteers at 37 °C for 1 h. The cytotoxicity studies of the developed CNC were carried out by mixing the blood samples of healthy human volunteers with various concentrations of CNC followed by separation of plasma. Free hemoglobin was measured by mixing the separated plasma with drabkins reagent followed by measuring the absorbance at 540 nm [112]. It was concluded that that the developed CNC did not exhibit any significant toxicity as compared to the reference sample [112]. The bacterial nanocellulose was evaluated for cytotoxicity, genotoxicity, and mutagenicity by Coelha et al. The lenses prepared with the bacterial nanocellulose showed a high cell viability similar to that of the nanocellulose; however, low cell viability was observed in BC-QCD-DS (Bacterial Cellulose + Chitosan + Cyclodextrin + Diclofenac), BC-Q-CD-CP (Bacterial Cellulose + Chitosan + Cyclodextrin + Ciprofloxacin) as compared to nanocellulose. Genotoxicity was evaluated by a Kruskal–Wallis test followed by Dunn’s test for DNA % in the tail and tail moment. Studies showed that that BC-HCD -DS and BC-HCD have significant genotoxic effects (*p* > 0.05) [277]. The nuclear division index (NDI), frequency of micronucleus (FMN), and frequency of binucleate cells with a micronucleus (FBMN) were used to evaluate the cytokinesis block micronucleus assay (CBMN) for mutagenicity of the lenses. NDI studies showed that there is a significant difference between BC-H-CD and BC-H-CD-DS. In FBMN studies, there was no significant difference observed between the lenses and negative control. FMN was observed only in the positive control, indicating that the developed lenses do not have any mutagenic effects [277]. A similar study conducted by Gwak et al. evaluated the cytotoxic effects of cellulose nanofiber on the fibroblast (HDF-α) and keratinocyte cells (HaCaT). The result of the cytotoxicity showed that the CNF significantly inhibited the cell growth up to 313 µg/mL and 156 µg/mL in HDF-α and keratinocyte cells, respectively. The irritability studies done on the cornea model proved that the developed CNF was safe and had no irritation [278]. Since the ocular tissue is highly sensitive, it is of utmost important to establish the safety and toxicity of the ophthalmic formulations containing cellulosic polymers.

## 12. Conclusions

Overcoming the solubility and bioavailability issues of drugs for ocular delivery is a challenging task, which creates a need to explore novel polymers and adopt recent technologies. However, it is important to consider the safety and toxicity issues while selecting the drugs, polymers, and other excipients. Various polymers have proved to be of great assistance for the development of ocular delivery systems, out of which cellulose and its derivatives have shown promising results. Numerous studies have shown the capability of cellulose as a biocompatible polymer, proving it as a versatile and multifunctional polymer. Cellulose derivatives have shown their potential for increasing the solubility and bioavailability of ophthalmic drugs. The nanocomposites of cellulose and its derivatives have shown superior effects over other polymers for ocular delivery, thus making cellulose a suitable candidate for ocular drug delivery systems.

## 13. Future Prospects

Drug absorption and bioavailability enhancement by various derivatives of cellulose have been extensively researched and their efficiency has been established in ocular drug delivery systems. However, the cellulosic derivatives may suffer from limitations such as blurred vision, eye irritation, and redness in sensitive patients when used at higher concentrations. Therefore, there is a need for further research involving the modification of cellulose that can overcome these limitations. More clinical trials are needed to establish the efficacy of various cellulose derivatives in ocular drug delivery. Modifications such as nanosizing and surface modification of the cellulose are needed to be explored, but as nanosizing of the cellulose may have disadvantages, such as cytotoxicity, mutagenicity, and genotoxicity, there is a further need to study the ocular toxicity and safety associated with the modifications.

## Figures and Tables

**Table 1:** Cellulosic polymer structure, properties, and application.

Cellulosic Polymer	Structure/Modification	Properties	Application	Ref
Cellulose	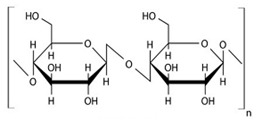	Sustainable natural polymer, good mechanical properties	Viscosity enhancer, water binding ability, adhesiveness	[111]
HPMC	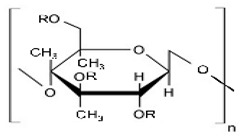	Biodegradable, biocompatible material, transparency, and rheological properties	Improve bioavailability and efficacy, prolonged retention.	[124]
CMC	R=H, CH3, CH_3_CH(OH)CH_2_ 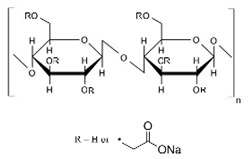	Biocompatible, biodegradable, non-toxic and water-soluble	Synergistic effects for dry eye with less blur vision and stickiness when blinking.	[125]
MC	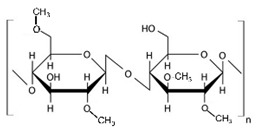	Water-soluble, non-toxic, tasteless, and odorless, LCST polymer	Prolonged residence time, Bio adhesion.	[126]
EC	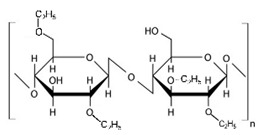	Linear, non-toxic, non-swellable polysaccharide, insoluble in water	Enhanced corneal resistance time, extended or control release, retard drug release	[127]
HEC	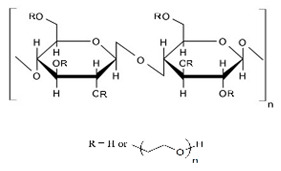	Biocompatible, biodegradable, hydrophilic, low toxic, and non-immunogenic	Sustained release and improve ocular performance	[128]
HPC	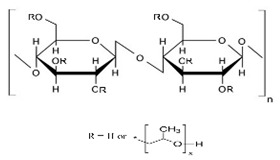	Highly plastic and hydrophobic compare to other cellulose ether derivatives	Increases the ocular residence time of drug	[129]
CA	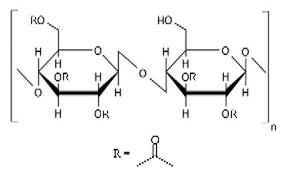	Non-toxic, non-irritant, and biodegradable polymer, it’s heat resistant and less hygroscopic	Sustain the release, prolong the residence time of the drug	[130]
CAP	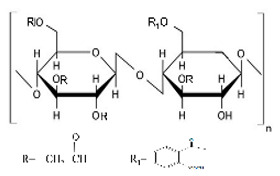	Hygroscopic to moisture and dissolve in GI fluid	Prolonge residence time and control permeation of drug	[105]

**Table 2:** Application of MC in ocular drug delivery.

Cellulosic Polymer	Drug Used	Application of Polymer	Effect on Drug Property	Ref
MC	Betaxolol hydrochloride	Sustain release upto 43% in 4 h	Enhanced AUC and MRT that is 47.10 ± 3.38 µL·h·g^−1^ and 5.17 ± 1.28 h compared to commercial preparation one 28.95 ± 4.12 µL·h·g^−1^ and 2.20 ± 0.06 h	[132]
	Sunitinib malate	Shear thinning agent, Zero shear prior injection	Enhanced injectability of MP, high retention of MPs after injection, reduced injection related inflammation, overall extended release	[133]
	Tranilast	Prolonged residence time	Enhanced efficacy of inflammation preventive effect of TL-NPs containing 0.5 or 1.5% MC, TNF-α and NO level was 50 pg/mg protein and 20 nM that is lowest in TL-MMC	[134]
	Pilocarpine nitrate	Bio-adhesion	Enhanced bioavailability due to bioadhesive nature of polymer	[135]

**Table 7:** Application of CMC in ocular drug delivery.

Cellulosic Polymer	Drug Used	Application of Polymer	Effect on Drug Property	Ref
CMC	Voriconazole	Improved residence time and sustained release upto 8 h and bioavailability	Mucoadhesive property of CMC enhanced the penetration of drug to cornea	[172]
	Voriconazole	Improve bioavailability	Enhanced in Cmax and tmax of in situ gel was 2203 ng/mlafter 1 h and increase in ocular bioavailability was seen by 5.91 fold greater AUC	[189]
	CelAgrip	Prolonged drug effect, film is transparent and non-irritating	Enhanced release upto 13.6 fold, increase in concentration of CMC cause reduction in release that is prolonged release	[190]
	Beclomethasone dipropionate	Bioavailability enhancement	Transcorneal permeation parameter P_app,_ flux and AUC_0–10h_ was enhanced by 4, 5.8 and 5.5 fold	[191]
	Mistletoe	Improved symptoms of dry eye	Symptoms of ocular surface was improved in pateints treated with mistletoe combineded CMC	[192]
	CMC and HPMC	For dry eye	OSDI and schirmer I test was 23.48, 22.75 ± 3.04	[193]
	Chloroquine	Synergistic effect for dry eye	After 3 month OSDI was 18.36 ± 4.03 bettter in groups taken chloroquin and CMC	[194]
	CMC and glycerin	Artificial tear formulation	CMC-GLY gel was found to be non inferior than CMC alone as OSDI and all parameter was significant	[195]
	CMC and hyaluronic acid	synergistic effects for dry eye	CMC-HA as found non inferior to CMC alone as all parameters was significant such as OSDI was −16.0 ± 16.1 and −16.9 ± 17.5	[196]
	CMC and hyaluronic acid	Synergistic effects for dry eye with less blur vision and stickiness when blinking	CMC and HA produced low shear viscosity while combined form had high shear viscoelasticity which remained unaffected therefore it was minimizing blurness and stickiness during blinking	[197]
	Vitamin-A	Enhances the stability and encapsulation	NC.CMC enhanced the stability of vegetable oil-water emulsion and ensured encapsulation of vitamin A in oil phase	[198]
	Flaxseed oil, trehalose and castor oil	Enhance emulsification of artificial tear	Improved baseline in OSDI score, ocular surface staining, and TBUT	[199]

**Table 8:** Patents on various cellulosic polymers used for ophthalmic formulations.

Title	Patent Number	Date of Publication	Inventors	Ref
Ophthalmic formulations providing durable ocular lubrications	WO 2020/123362 A1	18 June 2020	Willis, timothy, stone, ralph	[200]
Ophthalmic formulations, methods of manufacture, and methods of using the same	EEP2437602A1	11 April 2012	Matthew Jonathan Chapin, George Minno, jakie nice, George W, Ousler Lii, mark barry Abelson.	[201]
Dry eye treatment	US 9,044,388 B2	2 June 2015	Donald R korb, Chris J brancewicz. US	[202]
Methods of diagnosing and treating dry eye syndrome and compositions for treating a human eye	US 2020/0164013 A1	28 May 2020	Cott whitcup, laguna hills, orest olejnik, reno, michael garst.	[203]
Compositions providing improved eye comfort	US 2019/0060385 A1	28 February 2019	Mingqi bai, Ophthal Holeva.	[204]
Artificial tear emulsion	US 9,801,899 B2	31 October 2017	Claude claret, martin claret, Nicola amprecht weissenborn	[205]
Ophthalmic compositions	US 2013/0345185A	26 December 2013	Ashim k.mitra, Poonam R.velagaleti, Subramanian Natesan.	[206]
Ophthalmic compositions	EP2249871A2	17 November 2010	Arindam Halder, Ajay Jaysingh Khapade, Subhas Balaram Bowmick	[207]
Topical formulations and uses thereof	US 2020/0009137 A1	9 January 2020	Sidney L. Weiss.	[208]
Compositions and methods for treating ocular disorders	US 10,799,481 B1	13 October 2020	Tina de vries, David Jacobs.	[209]
Ophthalmic solution for artificial tear	US005591426A	7 January 1997	Henry P dabrowski, anil salpekar, o willian lever.	[210]

**Table 9:** Application of EC in ocular drug delivery.

Cellulosic Polymer	Drug Used	Application of Polymer	Effect on Drug Property	Ref
EC	Acetazolamide	Retard drugs release	Optimized drug:polymer ratio was 2:1 which had higher %EE (82.02 ± 2.5)	[216]
	Diclofenac sodium	Enhanced corneal resistance time, extended or control release upto 10% in 12 h at drug polumer ratio of 1/10	Drug release was 10% in 12 h with AUC and MRT 89.8 ± 24.6 µg h/mL and 7.22 ± 0.93 h	[217]
	Timolol maleate	Control release	NP 1:3 showed sustained release for more than 168 h and reduction in IOP from baseline for more than 192 h	[218]
	Timolol maleate	Sustain release of drug for ocusert	Drug EE%, content uniformity, and drug release was 94 to 98%, 93.1 ± 0.264 to 98.00 ± 0.321%, and 83.42 ± 0.35% for 12 h	[219]
	Dexamethasone	High encapsulating efficacy and loading capacity	Ethyl cellulose NP had more drug loading capacity (1.4 to 2.2%), slower drug release than conventional cream.	[220]

**Table 10:** Application of HEC in ocular drug delivery.

Cellulosic Polymer	Drug Used	Application of Polymer	Effect on Drug Property	Ref
HEC	Dorzolamide	Sustained release, decrease dosing frequency	Reduction in IOP for 2 week an drug also showed neuroprotective effect	[68]
	Ketorolac tromethamine	Effective carrier for delivery of the drug	Enhanced EE% and drug loading was 45.4 and 36.3%	[224]
	Azithromycin	Sustained release and improve ocular performance	Enhanced EE% up to 62.167 ± 0.07% and prolonged drug release for 121 h	[225]
	Betaxolol	Prolongation of pre-corneal retention and enhanced relative bioavailability	Enhanced encapsulation efficiency (69 ± 5%) and relative bioavailability (280 and 254.7%)	[226]
	Phenylephrine and tropicamide	Extended-release	Enhanced force of bioadhesion (5688 mpa) and mucoadhesive property 172.75 ± 50.81 pa and enhanced bioavailability	[227]
	Povidone-iodine	Additive effect for feline herpesvirus type1 and C Felis.	Enhanced relative cell viability up to 52.7 ± 2.1% *v*/*v* and significantly reduced C. felis growth	[228]
	Cysteamine	Viscosity enhancer	Enhanced drug released for 45–50 min, and combined with bioadhesive nature of gel results in enhanced bioavailability	[229]
	Oxidized hydroxy ethyl cellulose	Safe development, fashionable, and PH detectable contact lenses	Non-cytotoxic with excellent photochromic property of 2 × 10^−4^ and transparency up to 93% with compression strength 8.5 MPa	[63]

**Table 11:** Application of HPC, CA, and CAP in ocular drug delivery.

Cellulosic Polymer	Drug Used	Formulation	Application of Polymer	Effect on Drug Property	Ref
HPC	Timolol maleate	Instantly soluble solid eye drops	Increases the ocular residence time of drug	Enhanced corneal residence time by interacting with cornea because of mucoadhesive property of HPC	[129]
CA	Betaxolol hydrochloride	Silicone hydrogel based contact lenses	Sustained release up to 66% in 72 h, prolonged residence time of the drug	Enhanced bioavailability up to 50%, initial high burst release of 24.5 ± 5.2 µg/mL (to achieved therapeutic concentration) than decreased in release up to 1.7 ± 1.0 µg/mL at 6 h (sustained release)	[130]
CAP	Ibuprofen	Nanoparticle particle dispersed gel	Prolonged residence time and control permeation of drug	In vitro drug release was 70% at 8 h	[105]

## Data Availability

Data sharing not applicable.

## Abbreviations

BCS	Biopharmaceutics classification system
ODDS	Ocular drug delivery system
API	Active pharmaceutical ingredient
HPMC	Hydroxypropyl methylcellulose
CMC	Carboxymethyl cellulose
EC	Ethyl cellulose
MC	Methyl cellulose
HEC	Hydroxyethyl cellulose
HPC	Hydroxypropyl cellulose
CA	Cellulose acetate
CAP	Cellulose acetate phthalate
PVP	Polyvinyl pyrrolidone
PVA	Polyvinyl alcohol
CS	Chitosan
